# Quasi-Homoepitaxial Junction of Organic Semiconductors: A Structurally Seamless but Electronically Abrupt Interface between Rubrene and Bis(trifluoromethyl)dimethylrubrene

**DOI:** 10.1021/acs.jpclett.1c03094

**Published:** 2021-11-18

**Authors:** Kana Takahashi, Seiichiro Izawa, Naoya Ohtsuka, Atsuto Izumiseki, Ryohei Tsuruta, Riku Takeuchi, Yuki Gunjo, Yuki Nakanishi, Kazuhiko Mase, Tomoyuki Koganezawa, Norie Momiyama, Masahiro Hiramoto, Yasuo Nakayama

**Affiliations:** † Department of Pure and Applied Chemistry, Tokyo University of Science, 2641 Yamazaki, Noda 278-8510, Japan; ‡ Institute for Molecular Science, National Institutes of Natural Sciences, and SOKENDAI, 5-1 Higashiyama, Myodaiji, Okazaki, Aichi 444-8787, Japan; § Institute for Materials Structure Science, High Energy Accelerator Research Organization (KEK) and SOKENDAI, Tsukuba 305-0801, Ibaraki, Japan; ∥ Industrial Application Division, Japan Synchrotron Radiation Research Institute (JASRI), Sayo-gun 679-5198, Hyo̅go, Japan; ⊥ Division of Colloid and Interface Science, Tokyo University of Science, Noda 278-8510, Japan; # Research Group for Advanced Energy Conversion, Tokyo University of Science, Noda 278-8510, Japan; ¶ Precursory Research for Embryonic Science and Technology (PRESTO), Japan Science and Technology Agency (JST), 4-1-8 Honcho, Kawaguchi, Saitama 332-0012, Japan

## Abstract

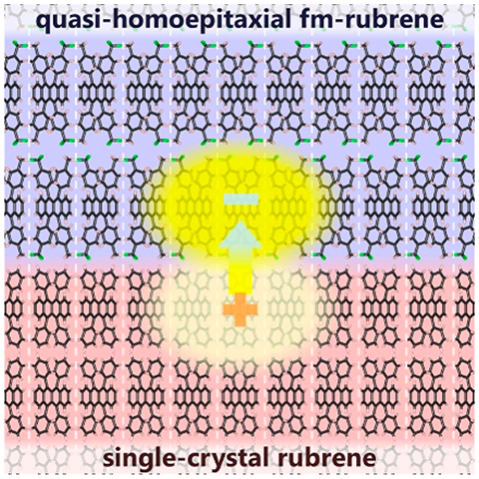

Single-crystalline organic semiconductors exhibiting band transport have opened new possibilities for the utilization of efficient charge carrier conduction in organic electronic devices. The epitaxial growth of molecular materials is a promising route for the realization of well-crystallized organic semiconductor p–n junctions for optoelectronic applications enhanced by the improved charge carrier mobility. In this study, the formation of a high-quality crystalline interface upon “quasi-homoepitaxial” growth of bis(trifluoromethyl)dimethylrubrene (fmRub) on the single-crystal surface of rubrene was revealed by using out-of-plane and grazing-incidence X-ray diffraction techniques. Ultraviolet photoelectron spectroscopy results indicated abrupt electronic energy levels and the occurrence of band bending across this quasi-homoepitaxial interface. This study verifies that the minimization of the lattice mismatch enhances the crystalline qualities at the heterojunctions even for van der Waals molecular condensates, potentially opening an untested route for the realization of high-mobility organic semiconductor optoelectronics.

Organic semiconductor electronics are attracting attention as next-generation optoelectronic devices because of their flexibility and low production costs. These merits are rooted in their solid-state nature, in which the organic semiconductor materials are constructed with relatively weak van der Waals interactions. In terms of crystal growth, this causes distinct characteristics of the organic semiconductors compared with their inorganic counterparts; one prominent example is polymorphism or tolerance in energies for various molecular packing structures because of shallow potential minima. For molecular thin films, this character leads to a tolerance for lattice mismatch to the substrates. Organic semiconductor molecules are therefore able to form uniformly oriented crystalline overlayers even on substrates with completely different lattice constants and symmetries, which is termed “van der Waals epitaxy” or “weak epitaxy”.^1−3^ Highly ordered intermolecular heterojunctions have been realized in this route, for which one can find detailed descriptions of the fundamental concepts and potential applications in several review papers.^4,5^

On the other hand, the weakness of the van der Waals interactions results in a drawback of organic semiconductor materials: poor charge carrier mobility. However, this problem can be addressed with the use of molecular crystals that realize “band transport”. Indeed, a number of molecular species have been reported to exhibit mobility exceeding that of amorphous silicon in single-crystalline field-effect transistor devices composed of these materials.^6−8^ In the case of optoelectronic devices such as organic photovoltaics, conventional architectures are composed of p-type (donor) and n-type (acceptor) materials of disordered condensates,^9,10^ and thus making use of the highly efficient charge carrier transport of single-crystalline structures may offer another possible concept to improve the device performance.^11−15^ In this context, epitaxial growth of organic semiconductors is a reasonable approach for obtaining crystalline p–n junctions through self-assembly. In previous studies, highly ordered heteroepitaxial interfaces of uniformly aligned n-type molecular materials formed on single crystals of p-type organic semiconductors have been attained.^16−21^ Moreover, it has been confirmed that single-crystalline thin films with extremely good crystallinity can be obtained by homoepitaxy,^22−24^ where molecules are deposited on single-crystal substrates of the same molecular species. This methodology enables the realization of p–n homojunctions of a monolithic organic semiconductor single crystal by accurately controlled impurity doping.^25^ Nevertheless, it is still unclear whether the electrostatic fields built in such gradual p–n homojunctions of doped organic semiconductor single crystals are too moderate to impact molecular excitons with strong Coulombic attractions.^14^

In this study, a well-ordered molecular interface of an organic semiconductor bis(trifluoromethyl)dimethylrubrene (fmRub) deposited on a rubrene single crystal (RubSC) substrate was elucidated. RubSC is a representative p-type material with a very high charge carrier mobility for organic semiconductors,^26,27^ and fmRub is a derivative of rubrene that exhibits ambipolar transport behavior with comparably high mobilities for both holes and electrons.^28^ The electronic basis for the prominent transport characteristics of RubSC, i.e., valence bands (VBs) with wide energy dispersion, has been experimentally verified,^29−31^ and widely dispersed electronic bands have also been predicted for fmRub by theoretical calculations.^32^ In terms of the crystallographic structures, these two species have very similar lattice constants in high-mobility planes, which is expected to fulfill the desired conditions for epitaxial growth with good crystallinity. In fact, the “quasi-homoepitaxial” growth of fmRub on the RubSC surface was successfully demonstrated by surface X-ray diffraction (XRD) measurements. Additionally, the electronic structure at this well-ordered interface was probed by using ultraviolet photoelectron spectroscopy (UPS), and the results indicated the occurrence of band bending presumably due to a small portion of electron transfer from RubSC to fmRub.

RubSC samples were prepared by using a physical vapor transport technique in a purified nitrogen stream, where “sublimed grade” (99.99% purity) source materials purchased from Sigma-Aldrich were used as received. Plate-shaped crystals were selected, and individual crystals were placed on bare and Au-coated silicon wafer pieces for XRD and photoemission experiments, respectively. For the latter, the crystals were surrounded by a conductive silver paste to ensure good electrical contact.^33,34^ FmRub was synthesized as described in Figure S1 and was deposited in vacuo onto the RubSC substrates at room temperature (RT). The deposition rate was set at 3 pm/s, which was monitored by using quartz microbalances. The surface morphology of the sample was observed by atomic force microscopy (AFM) and is represented in Figure S2.

The surface crystal structures were investigated by using out-of-plane XRD and grazing-incidence X-ray diffraction (GIXD) at BL19B2 of SPring-8 equipped with a six-circle diffractmeter 5021 (Huber). The X-ray wavelength was set to 1.00 Å, and the measurements were conducted under ambient conditions. GIXD measurements were employed by using a two-dimensional (2D) X-ray detector PILATUS-300K (for 2D-GIXD) or a NaI scintillation counter with doubled slits for the acquisition of spot profiles with higher precision (the slit widths were set at 0.2 mm unless otherwise noted). The X-ray glancing angle was set at 0.12° from the surface for GIXD measurements. The 2D detector was placed 173.3 mm from the in-plane sample rotation center facing perpendicular to the X-ray incident direction for the 2D-GIXD measurements, while the first and second slits were set 480 and 940 mm, respectively, from the sample rotation center for the high-precision measurements. The reported crystal structures for RubSC (*Cmca*, *a* = 26.86 Å, *b* = 7.193 Å, *c* = 14.433 Å, α = β = γ = 90° at 293 K^35^) and fmRub (*Pbcm*, *a* = 7.1443 Å, *b* = 14.0510 Å, *c* = 34.143 Å, α = β = γ = 90° at 123 K^32^), which are illustrated in Figure S3, were assumed for initial assignments of the diffraction patterns.

The electronic structure was measured by UPS at BL13B^36,37^ of KEK-PF using a SES-200 (Gammadata-Scienta) concentric hemispherical electron analyzer. The excitation photon energy was fixed at 30 eV, which was calibrated by using the Fermi edge positions of a metal plate excited by first- and third-order photons under identical monochromator settings.^38^ The work function of the analyzer was determined to be 4.48 eV. The measurements were performed under illumination of continuous-wave laser light (wavelength of 405 nm) to relieve the photoemission-induced sample charging with the assistance of photoconductivity.^29,39^ The vacuum level (*E*_vac_) position of the sample was determined from the lowest-energy cutoff position of the secondary electron emission under a negative sample bias (*V*_s_ = −5 V). Further details of the measurement conditions for the UPS are provided elsewhere.^40^ In this paper, the abscissae of the spectra are taken on a scale of the electron energy with respect to the Fermi level (*E*_F_). For the UPS experiments, fmRub was deposited on a RubSC sample in a stepwise manner under ultrahigh-vacuum conditions, and the measurements were conducted intermittently without breaking the vacuum. The measurements were performed with a normal emission geometry at RT.

An out-of-plane XRD profile of the fmRub/RubSC sample is shown in Figure 1a. The spiky peaks at *q*_*z*_ = 0.466, 0.932, and 1.399 Å^–1^ correspond to the 200, 400, and 600 reflections, respectively, for the (100) surface of the single-crystal rubrene, while the round peaks at *q*_*z*_ = 0.365, 0.73, 1.095, and 1.46 Å^–1^ can be attributed to the 002, 004, 006, and 008 spots, respectively, for the fmRub(001) surface. These results indicate that the fmRub overlayers grew in the (001) orientation on the RubSC(100) surface. The experimental *c*-axis height for fmRub was determined to be 34.37 Å.

**Figure 1:**
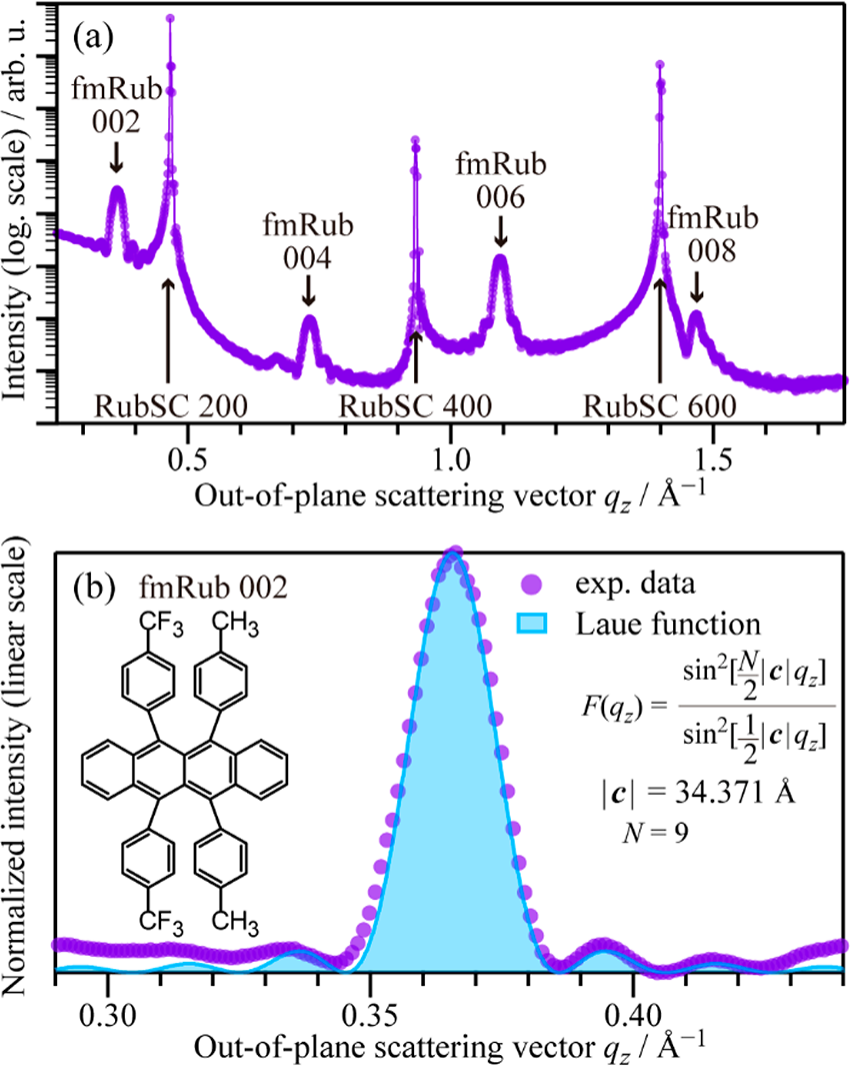
(a) Out-of-plane X-ray diffraction data for the fmRub(50 nm)/RubSC sample. (b) Close-up profile of the out-of-plane diffraction data at the fmRub 002 peak. The Laue function curve for nine units is also displayed in the light blue color.

Figure 1b shows a close-up profile of the fmRub(002) peak. The Laue oscillation accompanying the main diffraction spot indicates homogeneity in the thickness of the fmRub crystallites over the entire surface. From the spot width of the main peak and the periodicity of the Laue oscillation, the number of crystalline units, *N*, of the fmRub overlayer was estimated to be nine, as shown in Figure 1b, which corresponds to an out-of-plane crystalline size of about 28 nm. This value is smaller than the overlayer thickness of 50 nm, suggesting that the fmRub overlayer was not single crystalline but included domain boundaries and/or multiple crystallographic phases in the out-of-plane direction.^18^

Figure 2a shows a 2D-GIXD image of the fmRub/RubSC sample. This image was obtained by integrating 720 2D-GIXD data acquired during rotation of the in-plane sample azimuthal angle, ϕ, by 360°. In this image, diffraction spots assignable to the fmRub(001) surface were resolved in addition to those of the RubSC(100) surface, as shown in Figure 2a. An intense diffraction spot at around (*q*_*xy*_, *q*_*z*_) = (0.87 Å^–1^, 0.47 Å^–1^) and a weak spot at around (*q*_*xy*_, *q*_*z*_) = (0.88 Å^–1^, 0.37 Å^–1^) were attributed to the 202 spot of RubSC and the 102 or 022 spot of fmRub, respectively. As the surface lattice constant of fmRub(001) is very similar to that of RubSC(100), the fmRub 100 and 020 spots were overlapped by the RubSC 002 spot centered at (*q*_*xy*_, *q*_*z*_) = (0.87 Å^–1^, 0 Å^–1^). The spot intensities of these three *q* positions are plotted as a function of the in-plane azimuthal angle of the sample in Figure 2b–d, where the azimuthal angle at which the RubSC 002 spot appeared is hereafter defined as ϕ = 0. The diffraction conditions for the 202 and 002 spots of RubSC(100) were fulfilled at substantially the same ϕ angles in this measurement setup. As shown in Figure 2c, the fmRub-derived spots appeared at around ϕ = 0° and 180° (intense) and ϕ = 90° and 270° (weak). The fact that these diffraction spots appeared only at specific ϕ angles indicates epitaxial growth of fmRub on the RubSC(100) surface. It should be noted that whereas the 102 and 022 spots of fmRub yielded substantially the same *q* and thus could not be defined by the diffraction spot itself, the intense and weak spots could be assigned to the 022 and 102 diffractions, respectively, as a consequence of the ϕ angles where the 113 and  diffraction appeared (ϕ = 62°, 242° and ϕ = −65°, 115°, respectively). Therefore, the weak peak for (*q*_*xy*_, *q*_*z*_) = (0.87 Å^–1^, 0 Å^–1^) observed at ϕ = 90°, at which no substantial intensity was detected for the RubSC 202 position, was attributed solely to the 100 diffraction of fmRub.

**Figure 2:**
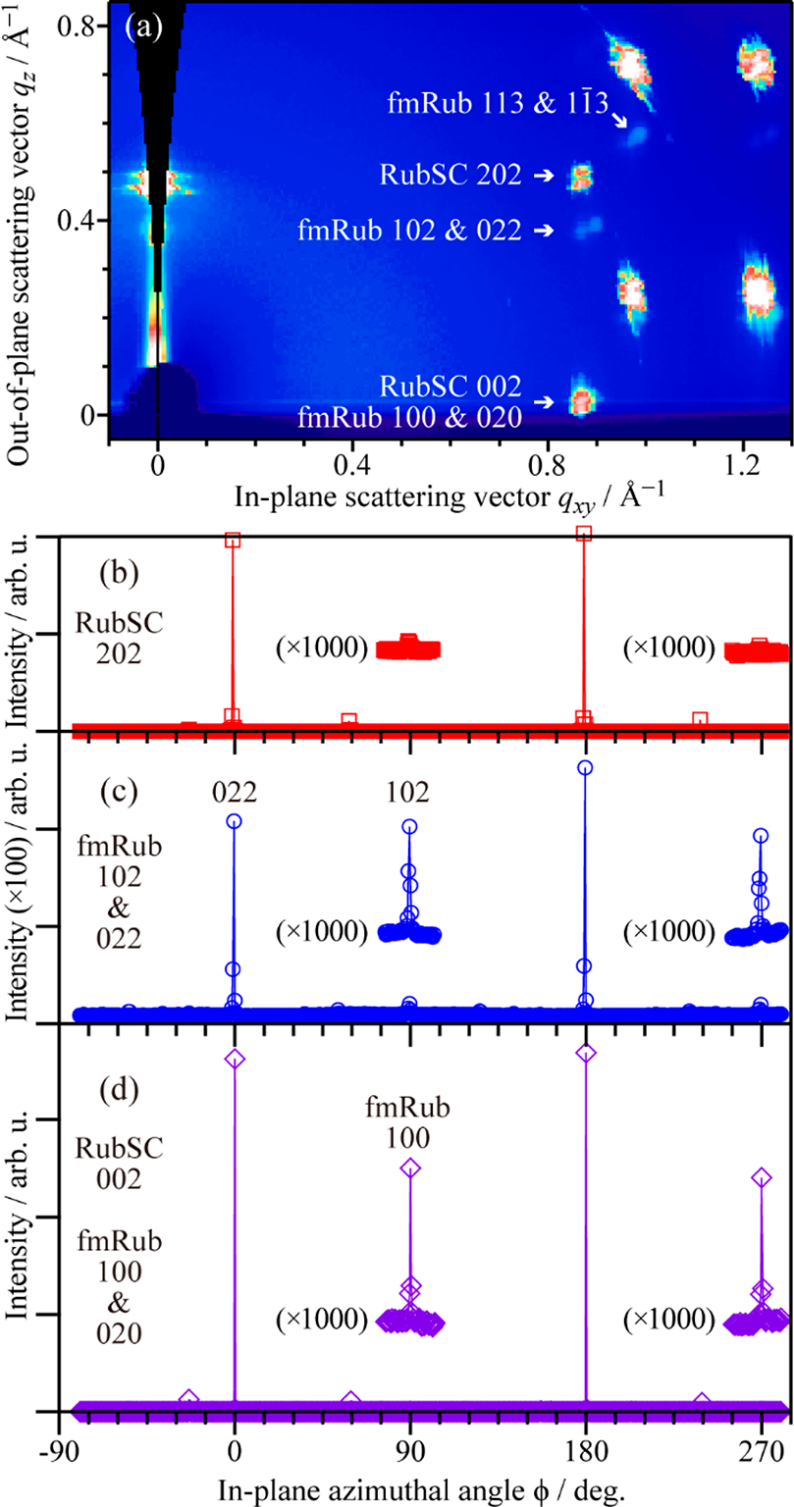
(a) ϕ-integrated 2D-GIXD image of the fmRub(50 nm)/RubSC sample. (b–d) Intensity distribution depending on ϕ at around (b) (*q*_*xy*_, *q*_*z*_) = (0.87 Å^–1^, 0.47 Å^–1^) attributable to the RubSC substrate (202 diffraction), (c) (*q*_*xy*_, *q*_*z*_) = (0.88 Å^–1^, 0.37 Å^–1^) attributable to the fmRub overlayers (102 and 022), and (d) (*q*_*xy*_, *q*_*z*_) = (0.87 Å^–1^, 0 Å^–1^) corresponding to both RubSC (002) and fmRub (100 and 020). The vertical scale of (c) is extended by a factor of 100 compared to those of (b) and (d).

The spot profiles of the 102 and 022 diffractions of fmRub and the 002 diffraction of RubSC were measured more accurately by using a scintillation counter. As shown in Figure 3a, the fmRub 022 spot had a maximal intensity at ϕ = −0.68°, which can be regarded as identical with the expected angle of ϕ = −0.699° for the fmRub(001) surface with its *b*-axis aligned along the *c*-axis of the RubSC(100) surface (Figure 3a, inset). The fmRub 102 diffraction spot was detected at ϕ = 89.28°, which also matched the expected position (ϕ = 89.271°) under the same assumption. These results confirmed that fmRub exhibited epitaxial growth on the RubSC(100) surface in an interlattice relationship where the *a*- and *b*-axes of fmRub aligned parallel to the *b*- and *c*-axes, respectively, of RubSC.

**Figure 3:**
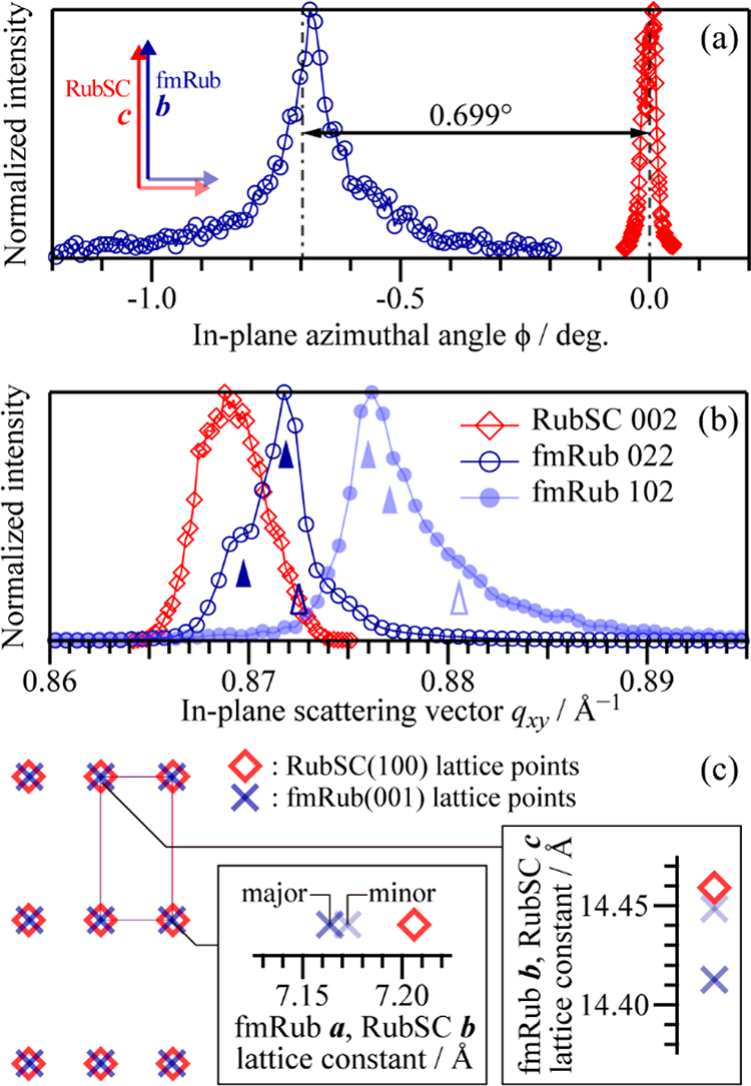
(a) Precise ϕ profiles of the RubSC 002 (red) and fmRub 022 (blue) diffraction spots. (b) Spot profiles in the *q*_*xy*_ direction of the RubSC 002, fmRub 022, and fmRub 102 diffractions. For the fmRub-derived spots, the peak positions of the three components for each are indicated as wedge marks (filled marks: sharp peaks; open marks: broad features). (c) Schematic drawing of the interlattice relationship between the epitaxial fmRub and the RubSC(100) substrate. The lattice constants were deduced based on the *q*_*xy*_ profiles shown in (b), where only the “sharp” components were taken into account for fmRub. The lattice mismatch for the primary (*a* of fmRub and *b* of RubSC) and secondary (*b* of fmRub and *c* of RubSC) axes are shown in the inset images, where the component with greater intensity for each spot was defined as the “major” component.

Figure 3b shows the *q*_*xy*_ profiles of these three diffraction spots. Whereas the RubSC 002 spot profile was obtained with the doubled-slit width of 0.5 mm, two fmRub-derived spots were measured in an identical measurement condition (the slit width of 0.2 mm). The *q*_*xy*_ position of the fmRub 022 spot was apparently smaller than that of the 102 spot for the present case, which is the opposite of the expected *q*_*xy*_ values (*q*_*xy*_ = 0.8943 Å^–1^ for 022 and *q*_*xy*_ = 0.8795 Å^–1^ for 102) from the bulk crystal structure. This implies that the thermal expansion coefficients of fmRub are significantly anisotropic for the *a*- and *b*-axes and/or the crystal lattice of the fmRub overlayer was slightly distorted from that of its bulk crystal. In addition, the fmRub-derived spots exhibited *q*_*xy*_ profiles suggesting the coexistence of multiple components rather than single peaks. Indeed, the fmRub 022 spot can be separated into two sharp peaks and one broad feature (as shown in Figure S4) whose positions are indicated as wedge marks in Figure 3b. The fmRub 102 peak can also be reproduced by using three components. The multiple peaks in the diffraction profiles suggest the concomitance of crystalline polymorphs with different lattice constants. For the two sharp fmRub 022 components, the center *q*_*xy*_ value of the most prominent peak was 100.32% of that of the RubSC 002 peak, and the minor component was located at the identical *q*_*xy*_ position to RubSC 002 (a deviation of <0.1%). Whereas the *q*_*xy*_ positions for the fmRub 102 components cannot be compared to the RubSC 010 diffraction because of the extinction rule, the *q*_*xy*_ ratios of the fmRub components correspond to an expected *q*_*xy*_ value derived from the RubSC 002 spot position, and the reported *b*/*c* ratio was in the range 100.4%–100.6%. On the basis of these GIXD results, the interlattice relationship between the epitaxial fmRub crystallites and the RubSC(100) surface was derived as illustrated in Figure 3c. For either possible polymorph, the in-plane crystalline lattice of fmRub coincided with that of RubSC with a lattice mismatch of <1%; in other words, fmRub exhibited “quasi-homoepitaxial” growth on the RubSC(100) surface.

It is worth noting that remarkable improvement in the crystallographic quality was implied for the present quasi-homoepitaxial fmRub on the RubSC in comparison to that of known heteroepitaxial molecular junctions. The total full widths at half-maximum (FWHM) including all the three components for the fmRub 102 and 022 diffraction peaks were 0.0053 and 0.0040 Å^–1^, respectively. Those widths correspond to in-plane mean crystallite sizes of 0.12 and 0.16 μm, respectively, which are already comparable to or even greater than those for RT-grown epitaxial C_60_ on the RubSC(100) surface.^19^ Furthermore, the FWHM of the sharpest component (0.0016 Å^–1^) corresponds to an in-plane mean crystallite size of 0.40 μm, which was 3 times greater than the case of C_60_/RubSC. The present results strongly suggest that minimization of the lattice mismatch at molecular junctions is also a decisive factor for the crystal quality even for the molecular semiconductors as van der Waals condensates. To further improve the in-plane crystallite size toward the ideally single-crystalline molecular junctions, the same approach as the cases for heteroepitaxial and homoepitaxial junctions can be taken; that is, an increase of the sample temperature for extending the surface diffusion constant of adsorbed molecules^18,19^ and a precise control of the deposition rate within a low level.^23^ Another possible way is the reduction of trapping sites for the adsorbates on the molecular crystal surfaces. Very flat surfaces with minimized molecular steps are favorable because these tend to accumulate the adsorbed molecules even for the organic semiconductor single crystals.^41,42^ In addition, since impurities should block the surface diffusion via modification of the adsorption potentials, protection of the single crystal surface from the exposure to the ambient condition to avoid (photo)oxidation^43−45^ as well as the use of extremely purified source materials^46^ may be potential measures for maximization of the crystallographic qualities of the molecular junctions.

In Figure 4, the evolution of the UPS spectra during stepwise deposition of fmRub on a RubSC sample is presented. For the bare RubSC sample (i.e., fmRub thickness of 0 nm), the onset position of the RubSC valence band (VB) was estimated to be at −0.55 eV from *E*_F_ based on linear extrapolation of the spectral slope, while the actual VB edge may be closer to *E*_F_ by ∼0.1 eV, considering a small tailing feature on the right side of the peak. A very slight change in the RubSC VB edge toward *E*_F_ by ∼0.03 eV was observed upon the deposition of fmRub, whereas the unchanged peak width suggests that the VB structures of RubSC were unaffected by the presence of fmRub.^41^ Further deposition of 3 nm thick fmRub concealed the peak derived from the RubSC VB; instead, a new peak attributable to the highest-occupied molecular orbital (HOMO) of fmRub emerged. The onset position of the peak was located at −1.39 eV from *E*_F_ for the 3 nm thick fmRub, and it shifted toward *E*_F_ as the fmRub thickness increased. The spectrum for the 1 nm thick fmRub can be regarded as a superposition of the photoemission from fmRub and the underlying RubSC,^20,47^ and the onset position of the spectral contribution of fmRub was estimated to be −1.45 eV as indicated in Figure 4. On the other hand, the work function (WF) of the bare RubSC sample was estimated to be 4.50 eV, which gradually increased as the fmRub thickness increased. The ionization energy of the present RubSC sample was determined to be 5.05 eV from the VB onset vacuum level positions, which is thought to be equivalent to literature values.^24,29,43^ Likewise, the ionization energy of the epitaxial fmRub was evaluated to be 5.89 ± 0.04 eV from the spectra of the 3, 10, and 20 nm thick fmRub samples, which is also consistent with a previous report.^28^

**Figure 4:**
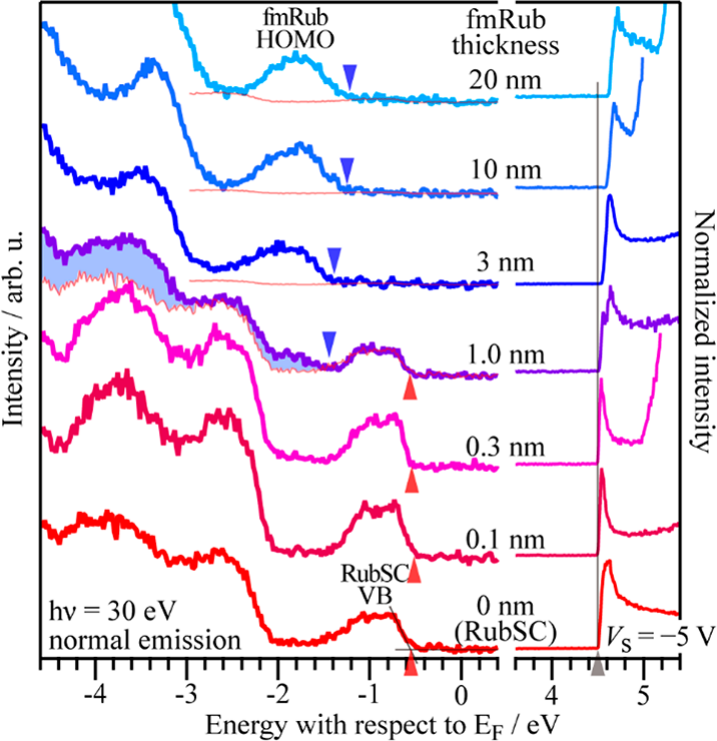
Evolution of the UPS spectra with increasing fmRub thickness on a RubSC sample. The left and right panels show the spectra in the valence electron and secondary electron regions, respectively, where the vertical scale of the latter is normalized by the secondary-electron cutoff (SECO) height for each curve. For the valence spectra for fmRub thicknesses of 1.0 nm and above, the hypothetical spectral contributions of the underlying RubSC are displayed in thin lines, and the contribution of fmRub is highlighted as shaded areas for the 1.0 nm thick sample. The onset positions estimated by a linear extrapolation (as given for the bare RubSC curve) are indicated by upward wedge marks for the RubSC VB and by downward wedges for the fmRub HOMO. The SECO position for the bare RubSC is displayed as a thin vertical line.

The UPS results are summarized in Figure 5a,b. The *E*_vac_ shifted upward upon the stacking of fmRub from the initial RubSC position of 4.50 eV (from *E*_F_) by 0.17 eV in total. This trend in the *E*_vac_ shift was similar to that of an epitaxial heterojunction of perfluorinated pentacene on pentacene single crystals (PnSCs)^20^ but was in contrast to those of cases of heteroepitaxial junctions of C_60_ and tetraazanaphthacene (TANC) on PnSCs, which had a constant *E*_vac_.^21,48^ The VB of RubSC also shifted slightly upward after the fmRub deposition; in other words, the *E*_F_ moved toward the VB, implying the occurrence of acceptor doping to RubSC.^23,24^ The suppression of the VB-derived peak of RubSC suggests the complete covering of the RubSC surface by the fmRub overlayer, at least at an fmRub thickness of 3 nm. It is noteworthy that this thickness was much greater than the probing depth (<1 nm) for UPS in the present measurement conditions^49^ and was equivalent to the *c*-axis lattice constant (3.44 nm), which consists of two molecular layers of fmRub. This implies that the quasi-homoepitaxial fmRub overlayers were formed in a layer-by-layer (or bilayer-by-bilayer) growth mode, which is in contrast to the C_60_/PnSC and TANC/PnSC cases.^16,21^ On the other hand, the HOMO of fmRub also shifted upward with an increase in fmRub thickness. In reverse, the fmRub HOMO, which was located at −1.22 eV from *E*_F_ for its bulk state, was bent downward by 0.1–0.2 eV as it approached the heterojunction with RubSC.

**Figure 5:**
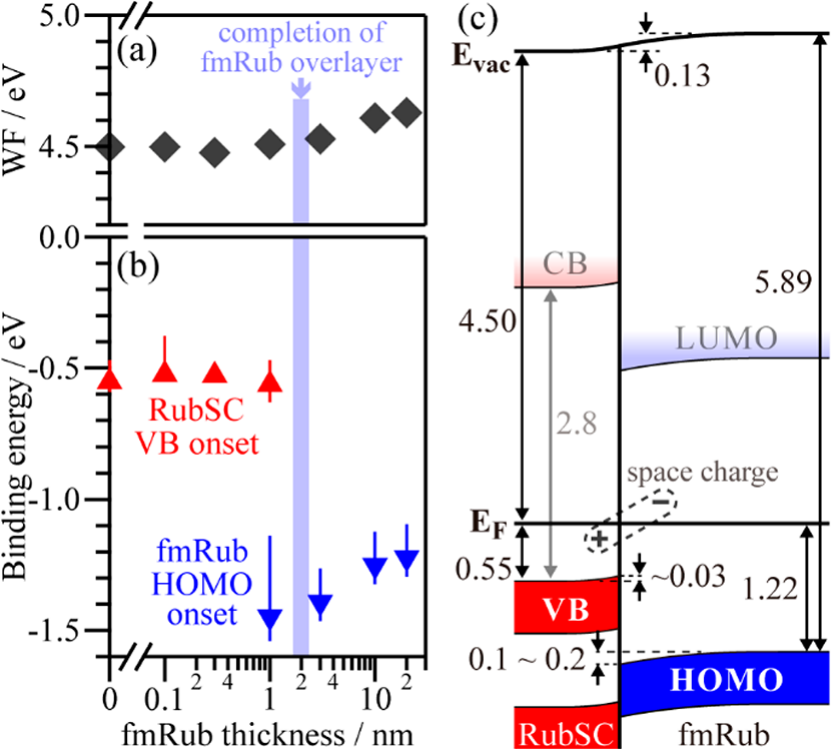
(a) Sample work function values (*E*_vac_ positions) plotted as a function of the fmRub thickness. (b) Onset energies of the RubSC VC (upward triangles) and fmRub HOMO (downward triangles) plotted as a function of the fmRub thickness. The error bars correspond to possible energy ranges, considering both the tailing features and ambiguities of the linear extrapolation. (c) Energy level diagram across the topical quasi-homoepitaxial junction of fmRub on formed the RubSC surface.

Figure 5c shows an energy level diagram across the quasi-homoepitaxial junction of fmRub and RubSC derived from the UPS results. In this diagram, the reported energy gap width of RubSC (2.8 eV^50^) was adopted to place the conduction band (CB) edge position of the RubSC side, and the same gap width was also assumed for fmRub.^32^ In addition, an assumption of constant ionization energy was adopted; that is, the VB of RubSC and the HOMO of fmRub were considered to be parallel to *E*_vac_. The extended energy shifts for the HOMO of fmRub and *E*_vac_, even after the completion of the fmRub overlayer, indicate the presence of band bending and thus of a space charge within the fmRub layers. Although the expected position for the lowest-unoccupied molecular orbital (LUMO) of fmRub was far above *E*_F_, the present results suggest the occurrence of electron transfer from RubSC to fmRub to some extent presumably via midgap states distributed around *E*_F_.^24^

The band bending that tends to accumulate both holes and electrons at the fmRub/RubSC junction seems to be disadvantageous for photovoltaic applications of conventional architectures where photogenerated charge carriers are removed in the direction perpendicular to the heterojunctions. However, it has been reported that band bending does not always alter the probabilities for charge carrier recombination,^51^ presumably because the potential distributions inside the “working” solar cells cannot be assumed in a straightforward manner from the energy level diagrams, as suggested by photoemission results obtained under the simulated sunlight illumination.^52^ On the other hand, considering that efficient charge carrier conduction for both molecules occurs within the crystalline layers (i.e., the *bc*-plane for RubSC and the *ab*-plane for fmRub), the band bending is presumed to induce small, if any, impacts on the charge carrier transport in the most conductive directions. Recently, organic photovoltaic devices with a “new concept” that utilizes the band transport of such high-mobility organic semiconductor materials in the lateral directions have been proposed,^15^ which may be a promising route for applications of this heterojunction. It is worth noting that the built-in electrostatic field of this band bending enables the 2D accumulation of both electrons and holes in the conductive crystalline planes along this quasi-homoepitaxial junction. This may be a favorable condition for organic light-emitting transistors or organic lasers that demand a high recombination rate and excellent charge carrier transport efficiencies.^53,54^

In summary, the crystal structure and electronic states of fmRub overlayers formed on RubSC were analyzed by using the surface XRD techniques and UPS. Quasi-homoepitaxial growth of the (001)-oriented fmRub overlayers, whose lattice mismatch was determined to be less than 0.05 Å and 0.02° to the RubSC(100) surface, was demonstrated. The mean crystallite size in the out-of-plane direction (ca. 28 nm) was less than the total overlayer thickness (50 nm), implying the presence of multiple crystalline domains. However, the in-plane mean crystallite size of no smaller than 120 nm indicated improved crystallinity of this quasi-homoepitaxial molecular junction in comparison to known heteroepitaxial junctions such as C_60_/RubSC. From the UPS results, an energy level diagram across the quasi-homoepitaxial junction was deduced. An upward band bending from the RubSC side to the fmRub was revealed by UPS, which was presumably caused by the occurrence of a slight electron transfer from RubSC to fmRub. In recent years, “wafer-scale” fabrication of organic single crystals has been realized by use of liquidus solutions,^55^ and even single-crystalline p–n heterojunctions of molecular semiconductors have been produced by simple solution processes in an extended scale.^56^ It is noteworthy that the present novel concept of molecular quasi-homoepitaxy enabling the concomitance of good crystallographic qualities and abrupt electronic energy level offsets at the molecular junctions is in principle applicable not only to the vacuum deposition schemes but also to the solution grown techniques toward low-cost and wide-area organic optoelectronic applications.

## Supporting Information Available

Synthesis of bis(trifluoromethyl)dimethylrubrene;^32,57^ atomic force microscopy (AFM) images of the fmRub/RubSC sample; crystal structures of rubrene and bis(trifluoromethyl)dimethylrubrene;^32,35,58^ peak separation for the *q*_*xy*_ profiles of fmRub 102 and 022 spots (PDF)
