# Supplementary material for: Quasi-Homoepitaxial Junction of Organic Semiconductors: A Structurally Seamless but Electronically Abrupt Interface between Rubrene and Bis(trifluoromethyl)dimethylrubrene
Source: J Phys Chem Lett. 2021 Nov 18;12(46):11430–7. doi: 10.1021/acs.jpclett.1c03094 (PMC8630783; doi:10.1021/acs.jpclett.1c03094)
Supplement: Supplementary file 1 — jz1c03094_si_001.pdf [file jz1c03094_si_001.pdf]

Supporting Information

Quasi-Homoepitaxial Junction of Organic Semiconductors: a Structurally Seamless but Electronically Abrupt Interface between Rubrene and Bis(trifluoromethyl)dimethylrubrene

Kana Takahashi[1], Seiichiro Izawa[2,3], Naoya Ohtsuka[2], Atsuto Izumiseki[2],
Ryohei Tsuruta[1,#], Riku Takeuchi[1], Yuki Gunjo[1], Yuki Nakanishi[1], Kazuhiko Mase[4],
Tomoyuki Koganezawa[5], Norie Momiyama[2], Masahiro Hiramoto[2],
Yasuo Nakayama[1,6,7,*]

1: Department of Pure and Applied Chemistry, Tokyo University of Science, Japan
2: Institute for Molecular Science, National Institutes of Natural Sciences, and SOKENDAI, Japan
3: Precursory Research for Embryonic Science and Technology (PRESTO), Japan Science and Technology Agency (JST), Japan
4: Institute for Materials Structure Science, High Energy Accelerator Research Organization (KEK) and SOKENDAI, Japan
5: Industrial Application Division, Japan Synchrotron Radiation Research Institute (JASRI), Japan
6: Division of Colloid and Interface Science, Tokyo University of Science, Japan
7: Research Group for Advanced Energy Conversion, Tokyo University of Science, Japan

# Present affiliation: Graduate School of Science and Technology, University of Tsukuba, Japan
* Author to whom correspondence should be addressed: nkym@rs.tus.ac.jp

Contents

S1. Synthesis of bis(trifluoromethyl)-dimethyl-rubrene 2
S2. Atomic force microscopy (AFM) images of the fmRub/RubSC sample 12
S3. Crystal structures of rubrene and bis(trifluoromethyl)-dimethyl-rubrene 13
S4. Peak separation for the q_{xy} profiles of fmRub 102 and 022 spots 15

S1. Synthesis of bis(trifluoromethyl)-dimethyl-rubrene

General Information

Infrared (IR) spectra were recorded on a Jasco FT/IR-460 plus using ATR. ^{1}H NMR spectra were recorded on a JEOL ECS-400 (400 MHz) spectrometer. Chemical shifts are reported in ppm from the solvent resonance or tetramethylsilane (TMS) as the internal standard ($CDCl_3$: referenced to TMS 0.00 ppm). Data are reported as follows: chemical shift, multiplicity (br = broad, s = singlet, d = doublet, t = triplet, q = quartet, dd = double doublet, m = multiplet), integration and coupling constants (Hz). ^{13}C NMR spectra were recorded on a JEOL ECA-600 (151 MHz) spectrometer. Chemical shifts are reported in ppm from the solvent resonance as the internal standard ($CDCl_3$: 77.0 ppm). ^{19}F NMR spectra were recorded on a JEOL ECS-400 (376 MHz) spectrometer. Chemical shifts are reported in ppm from $CF_3C_6H_5$ as external standard or internal standard ($CF_3C_6H_5$: –63.72 ppm). High-resolution mass spectra (HRMS) analysis (FAB) was performed on a JEOL JMS-700 with 3-nitrobenzyl alcohol as the matrix at the Instrument Center, Institute for Molecular Science.

Unless otherwise noted, all reactions were carried out under an atmosphere of standard grade nitrogen gas (oxygen <10 ppm) in flame-dried glassware with magnetic stirring. Purification of reaction products was carried out by column chromatography using silica gel 60 N (Merck: 0.040-0.063 nm). For thin-layer chromatography (TLC) analysis throughout this work, Merck precoated TLC plates (silica gel 60 F_{254} 0.25 mm) were used. Visualization was accomplished by UV light (254 nm), and with phosphomolybdic acid as indicators. Anhydrous THF, CH_2Cl_2, and ether were supplied from Kanto Chemical Co., Inc. as “Dehydrated solvent system”. Other solvents were supplied from Wako Pure Chemical Industries Ltd. and Kanto Chemical Co., Inc. as dehydrated solvents. Reagents were purchased from commercial suppliers and used without further purification. Other simple chemicals were analytical-grade and obtained commercially.

Synthesis of **S-1**[1)]

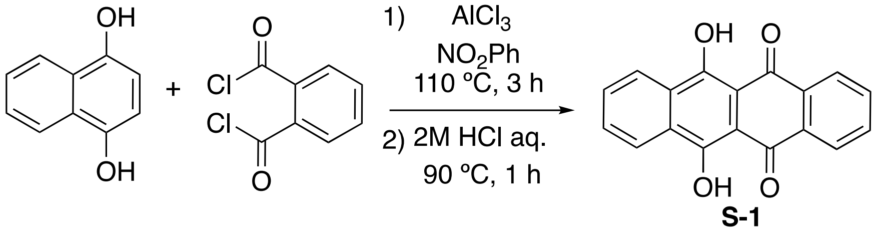

To a solution of 1,4-dihydoroxynaphthalene (1.60g, 10.0 mmol, 1.00 equiv.) in nitrobenzene (10.0 mL) was added $AlCl_3$ (3.33g, 25.0 mmol, 2.50 equiv.). Phthaloyl chloride (1.44 mL, 10.0 mmol, 1.00 equiv.) was added carefully dropwise to the mixture at room temperature. The mixture was warmed to 110 ºC and stirred for 3 h. After cooling to room temperature, 2M-HCl aq. (100 mL, 20.0 equiv.) was added to the mixture. The mixture was further stirred at 90 ºC for 1 h. The obtained suspension was filtered by Kiriyama. The residue was washed with H_2O (10 mL x 3) then EtOH (10 mL x 3), and dried under vacuo to give a **S-1** as red solid in 65% yield (1.88 g, 6.48mmol).

^{1}H NMR ($CDCl_3$ 400 MHz) δ 15.19 (brs, 2H), 8.51-8.48 (m, 4H), 7.86-7.82 (m, 4H).

Synthesis of **S-3**

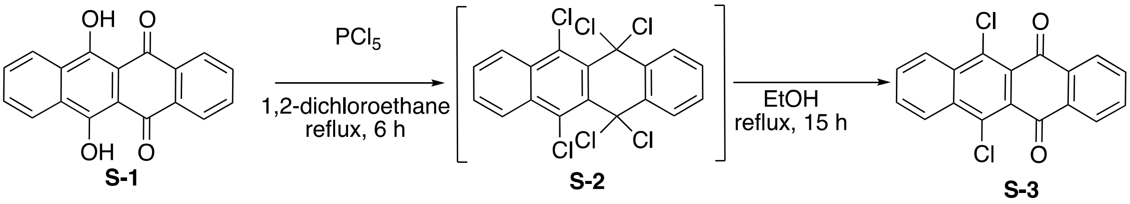

To a suspension of **S-1** (1.88 g, 6.48 mmol, 1.00 equiv.) in 1,2-dichloroethane (13.0 mL) was added PCl_5 (6.75 g, 32.4 mmol, 5.00 equiv.) one portion. The mixture was stirred at 130 ºC for 6 h. After cooling to 0 ºC, EtOH (10 mL) was slowly added to the mixture. The suspension was filtered by Kiriyama. The residue was washed with EtOH (5 mL x 3) and hexane (5 mL x 3) then dried under vacuo to give a **S-2**. **S-2** was used to next reaction without further purification.

The **S-2** was suspended in EtOH (10 mL). The suspension was stirred at 130 ºC for 15 h under air. After the mixture was cooled to room temperature, the suspension was filtered. The residue was washed with EtOH (5 mL x 3) then dried under vacuo to give a **S-3** as light brown solid in 81% yield (1.72 g, 5.24 mmol, 2 step yield from **S-1**).

^{1}H NMR ($CDCl_3$ 400 MHz) δ 8.76 (dd, J = 3.2, 3.2 Hz, 2H), 8.22 (dd, J = 3.4, 2.3 Hz, 2H), 7.87 (dd, J = 3.2, 3.2 Hz, 2H), 7.98 (dd, J = 3.2, 2.5 Hz, 2H).
^{13}C NMR ($CDCl_3$, 151 MHz) δ 182.7, 134.9. 134.0, 133.9, 130.9, 127.9, 127.4, 126.9.
IR (ATR) 2929, 1675, 1381, 1253, 917, 754, 712, 664 cm^{-1}.
HRMS (FAB) *m/z* Calcd for $C_{18}H_9Cl_2O_2$ ($[M + H]^+$): 326.9980. Found: 326.9980.

Synthesis of **S-4**[2)]

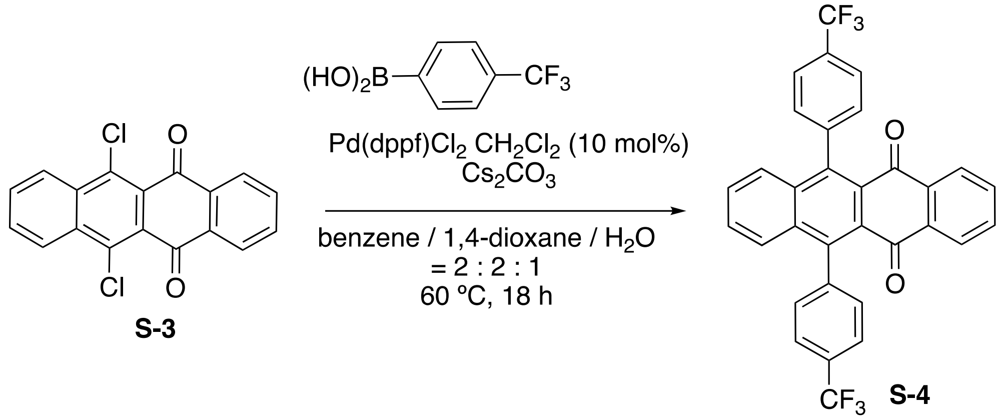

Benzene (35 mL), 1,4-dioxane (35 mL), and H_2O (18 mL) were charged to 300 mL three neck flask under nitrogen atmosphere. Nitrogen gas was bubbled through the solvents for 15 min. **S-3** (1.72 g, 5.24 mmol, 1.00 equiv.), 4-(trifluoromethyl)phenylboronic acid (2.99 g, 15.77 mmol, 3.00 equiv.), Cs_2CO_3 (6.86 g, 21.04 mmol, 4.00 equiv.), and $Pd(dppf)Cl_2 \cdot CH_2Cl_2$ (429 mg, 0.526 mmol, 10 mol%) were added. The mixture was warmed to 60 ºC and stirred for 18 h. After cooling to room temperature, H_2O (50 mL) was added to the mixture. The mixture was through celite pad and washed with CH_2Cl_2. The organic phase was separated. The aqueous phase was extracted with CH_2Cl_2 (20 mL x 3). The combined organic phases were washed with brine, dried over Na_2SO_4, and concentrated under reduced pressure after filtration. The crude product was purified by silica gel column chromatography (hexane/CH_2Cl_2 = 2:1 as eluent) to give a **S-4** as yellow solid in 59% yield (1.68 g, 3.09 mmol).

^{1}H NMR ($CDCl_3$ 400 MHz) δ 8.09-8.06 (m, 2 H), 7.86 (d, J = 7.8 Hz, 4 H), 7.22-7.69 m, 2H), 7.57-7.54 (m, 2 H), 7.47-7.40 (m, 6H).
^{19}F NMR ($CDCl_3$ 376 MHz) δ –62.05 (s, 6F).

Synthesis of **S-5**[2)]

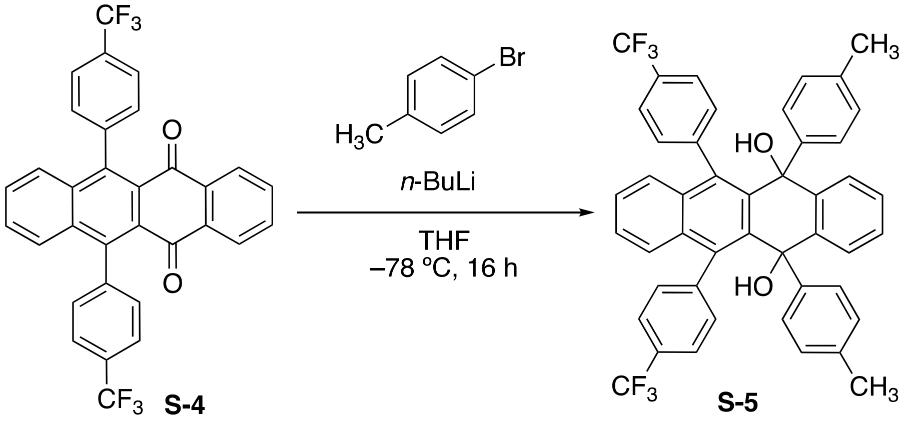

p-Bromotoluene (3.04 mL, 24.59 mmol, 8.00 equiv.) and THF (25 mL) were charged to a flame dried flask. After cooling to –78 ºC, *n*-BuLi (1.56 M in hexane, 14.7 mL, 23.36 mmol, 7.61 equiv.) was added dropwise. The mixture was stirred at –78 ºC for 30 min to afford a solution of 4-(methyl)phenyl lithium in THF.

To another 200 mL three neck flask was charged **S-4** (1.68 g, 3.09 mmol, 1.00 equiv.) and THF (25 mL). The solution was cooled to –78 ºC then the prepared solution of 4-(methyl)phenyl lithium in THF was added using cannula. The mixture was allowed to warm to room temperature, and stirred for 18 h. The mixture was quenched with saturated NH_4Cl aq. (50 mL) and extracted with Et_2O (20 mL x3). The combined organic phases were dried over Na_2SO_4, and concentrated under reduced pressure after filtration. The crude product was purified by silica gel column chromatography (hexane 100% to hexane/CH_2Cl_2 = 2:1 to CH_2Cl_2 100%) to give a **S-5** (mixture of diastereomer) as right brown solid in 97 % yield (2.18 g, 2.98 mmol *dr* = 75:25).

^{1}H NMR ($CDCl_3$ 400 MHz) δ 7.69 (d, *J* = 8.5 Hz, 0.7H, *minor*), 7.35 (d, *J* = 7.8 Hz, 2H *major*), 7.23-7.19 (m, 4.7H, *major and minor*), 7.13-7.08 (m, 1.4H, *minor*), 7.02 (d, *J* = 8.24 Hz, 2H *major*), 6.96-6.92 (m, 3.4H, *major and minor*), 6.88-6.79 (m, 14.8H *major and minor*), 6.24 (d, *J* = 8.24 Hz, 2H, *major*), 6.14 (d, *J* = 8.24 Hz, 0.7H *minor*) 3.90 (brs, 2H, *major*), 2.98 (brs, 0.7 H, *minor*), 2.28 (s, 6H, *major*), 2.27 (s, 2.1H, *minor*).
^{19}F NMR ($CDCl_3$ 376 MHz) δ –63.72 (s, 2.1F, *minor*), –63.83 (s, 6F, *major*).

Synthesis of fm-rubrene (**FmRub**)[2)]

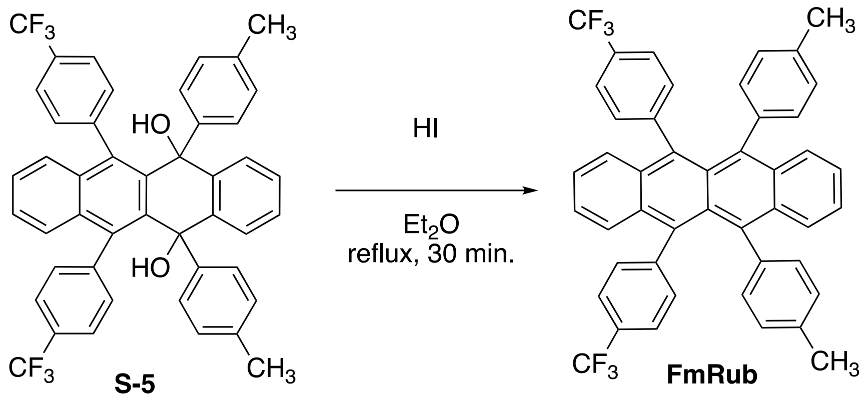

A solution of **S-5** (371 mg, 0.508 mmol, 1.00 equiv.) in Et_2O (20 mL) was warmed to 40 ºC. 55% purity of HI (555 μL, 4.06 mmol, 8.00 equiv.) was added to the refluxing solution then the mixture was stirred at 40 ºC for 30 min. After cooling to room temperature, the mixture was poured into 10% $NaHCO_3$ aq. (20 mL). The organic phase was separated, washed with sat $Na_2S_2O_3$ aq. (10 mL), dried over Na_2SO_4, and concentrated under reduced pressure after filtration. The obtained residue was washed with hexane and dried under vacuo to give a **FmRub** as red solid in 90% yield (320 mg, 0.457 mmol).

^{1}H NMR ($CDCl_3$ 400 MHz) δ 7.42 (dd, *J* = 3.7, 3.2 Hz, 2H), 7.32 (d, *J* = 8.2 Hz, 4H), 7.27-7.23 (m, 2H), 7.17-7.11 (m, 4H), 6.99 (d, *J* = 8.0 Hz, 4H), 6.86 (d, *J* = 8.0 Hz, 4H), 6.73 (d, *J* = 7.8 Hz, 4H), 2.37 (s, 6H).
^{19}F NMR ($CDCl_3$ 376 MHz) δ –62.35 (s, 6F).

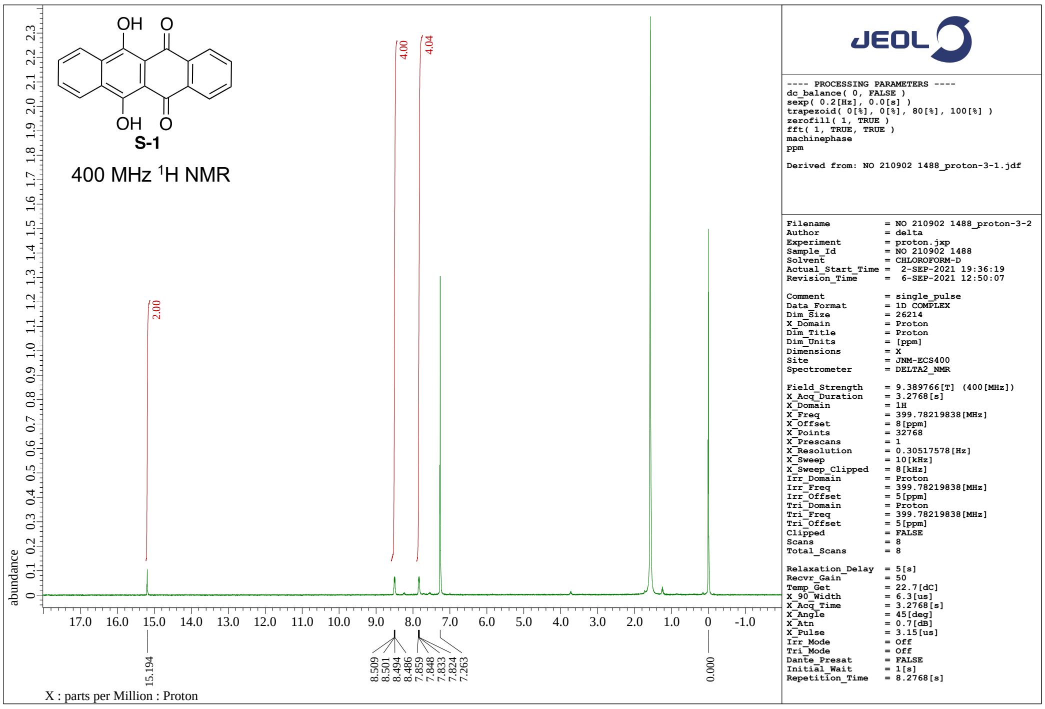
OH O
OH O
S-1
400 MHz 1H NMR
abundance
X : parts per Million : Proton
2.00
4.00
4.04
15.194
8.509
8.501
8.494
8.486
7.859
7.848
7.833
7.824
7.263
0.000
JEOL
---- PROCESSING PARAMETERS ----
dc_balance(0, FALSE)
sexp(0.2[Hz], 0.0[s])
trapezoid(0[%], 0[%], 80[%], 100[%])
zerofill(1, TRUE)
fft(1, TRUE, TRUE)
machinephase
ppm
Derived from: NO 210902 1488_proton-3-1.jdf
Filename = NO 210902 1488_proton-3-2
Author = delta
Experiment = proton.jxp
Sample_Id = NO 210902 1488
Solvent = CHLOROFORM-D
Actual_Start_Time = 2-SEP-2021 19:36:19
Revision_Time = 6-SEP-2021 12:50:07
Comment = single_pulse
Data_Format = 1D COMPLEX
Dim_Size = 26214
X_Domain = Proton
Dim_Title = Proton
Dim_Units = [ppm]
Dimensions = X
Site = JNM-ECS400
Spectrometer = DELTA2_NMR
Field_Strength = 9.389766[T] (400[MHz])
X_Acq_Duration = 3.2768[s]
X_Domain = 1H
X_Freq = 399.78219838[MHz]
X_Offset = 8[ppm]
X_Points = 32768
X_Prescans = 1
X_Resolution = 0.30517578[Hz]
X_Sweep = 10[kHz]
X_Sweep_Clipped = 8[kHz]
Irr_Domain = Proton
Irr_Freq = 399.78219838[MHz]
Irr_Offset = 5[ppm]
Tri_Domain = Proton
Tri_Freq = 399.78219838[MHz]
Tri_Offset = 5[ppm]
Clipped = FALSE
Scans = 8
Total_Scans = 8
Relaxation_Delay = 5[s]
Recvr_Gain = 50
Temp_Get = 22.7[dC]
X_90_Width = 6.3[us]
X_Acq_Time = 3.2768[s]
X_Angle = 45[deg]
X_Atn = 0.7[dB]
X_Pulse = 3.15[us]
Irr_Mode = Off
Tri_Mode = Off
Dante_Presat = FALSE
Initial_Wait = 1[s]
Repetition_Time = 8.2768[s]

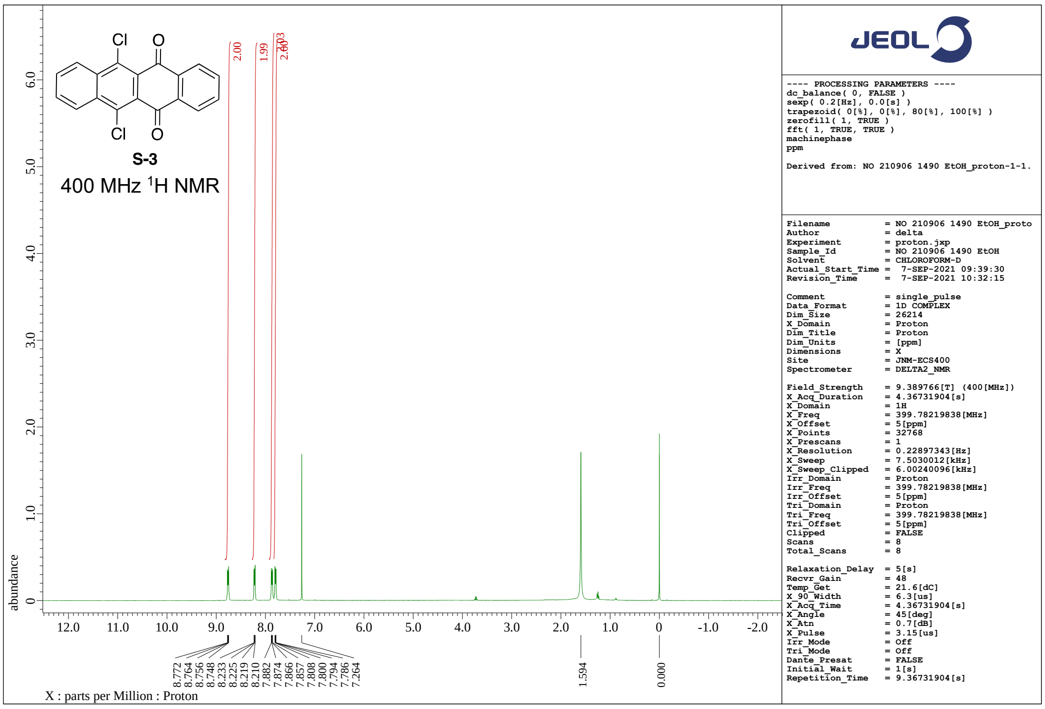
Cl O
Cl O
S-3
400 MHz 1H NMR
abundance
X : parts per Million : Proton
2.00
1.99
2.03
8.772
8.764
8.756
8.748
8.233
8.225
8.219
8.210
7.882
7.874
7.866
7.857
7.808
7.800
7.794
7.786
7.264
1.594
0.000
JEOL
---- PROCESSING PARAMETERS ----
dc_balance(0, FALSE)
sexp(0.2[Hz], 0.0[s])
trapezoid(0[%], 0[%], 80[%], 100[%])
zerofill(1, TRUE)
fft(1, TRUE, TRUE)
machinephase
ppm
Derived from: NO 210906 1490 EtOH_proton-1-1.
Filename = NO 210906 1490 EtOH_proto
Author = delta
Experiment = proton.jxp
Sample_Id = NO 210906 1490 EtOH
Solvent = CHLOROFORM-D
Actual_Start_Time = 7-SEP-2021 09:39:30
Revision_Time = 7-SEP-2021 10:32:15
Comment = single_pulse
Data_Format = 1D COMPLEX
Dim_Size = 26214
X_Domain = Proton
Dim_Title = Proton
Dim_Units = [ppm]
Dimensions = X
Site = JNM-ECS400
Spectrometer = DELTA2_NMR
Field_Strength = 9.389766[T] (400[MHz])
X_Acq_Duration = 4.36731904[s]
X_Domain = 1H
X_Freq = 399.78219838[MHz]
X_Offset = 5[ppm]
X_Points = 32768
X_Prescans = 1
X_Resolution = 0.22897343[Hz]
X_Sweep = 7.5030012[kHz]
X_Sweep_Clipped = 6.00240096[kHz]
Irr_Domain = Proton
Irr_Freq = 399.78219838[MHz]
Irr_Offset = 5[ppm]
Tri_Domain = Proton
Tri_Freq = 399.78219838[MHz]
Tri_Offset = 5[ppm]
Clipped = FALSE
Scans = 8
Total_Scans = 8
Relaxation_Delay = 5[s]
Recvr_Gain = 48
Temp_Get = 21.6[dC]
X_90_Width = 6.3[us]
X_Acq_Time = 4.36731904[s]
X_Angle = 45[deg]
X_Atn = 0.7[dB]
X_Pulse = 3.15[us]
Irr_Mode = Off
Tri_Mode = Off
Dante_Presat = FALSE
Initial_Wait = 1[s]
Repetition_Time = 9.36731904[s]

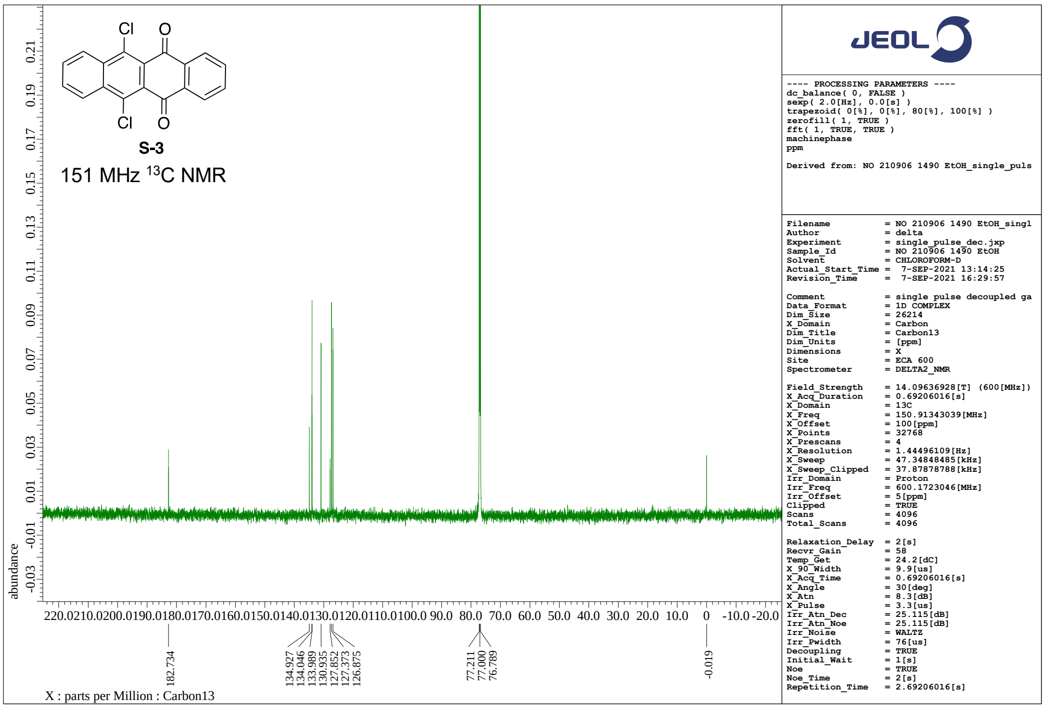
Cl
O
Cl
O
S-3
151 MHz 13C NMR
abundance
0.21
0.19
0.17
0.15
0.13
0.11
0.09
0.07
0.05
0.03
0.01
-0.01
-0.03
220.0 210.0 200.0 190.0 180.0 170.0 160.0 150.0 140.0 130.0 120.0 110.0 100.0 90.0 80.0 70.0 60.0 50.0 40.0 30.0 20.0 10.0 0 -10.0 -20.0
182.734
134.927
134.046
133.989
130.935
127.852
127.373
126.875
77.211
77.000
76.789
-0.019
X : parts per Million : Carbon13
---- PROCESSING PARAMETERS ----
dc_balance(0, FALSE)
sexp(2.0[Hz], 0.0[s])
trapezoid(0[%], 0[%], 80[%], 100[%])
zerofill(1, TRUE)
fft(1, TRUE, TRUE)
machinephase
ppm
Derived from: NO 210906 1490 EtOH_single_puls
Filename = NO 210906 1490 EtOH_singl
Author = delta
Experiment = single_pulse_dec.jxp
Sample_Id = NO 210906 1490 EtOH
Solvent = CHLOROFORM-D
Actual_Start_Time = 7-SEP-2021 13:14:25
Revision_Time = 7-SEP-2021 16:29:57
Comment = single pulse decoupled ga
Data_Format = 1D COMPLEX
Dim_Size = 26214
X_Domain = Carbon
Dim_Title = Carbon13
Dim_Units = [ppm]
Dimensions = X
Site = ECA 600
Spectrometer = DELTA2_NMR
Field_Strength = 14.09636928[T] (600[MHz])
X_Acq_Duration = 0.69206016[s]
X_Domain = 13C
X_Freq = 150.91343039[MHz]
X_Offset = 100[ppm]
X_Points = 32768
X_Prescans = 4
X_Resolution = 1.44496109[Hz]
X_Sweep = 47.34848485[kHz]
X_Sweep_Clipped = 37.87878788[kHz]
Irr_Domain = Proton
Irr_Freq = 600.1723046[MHz]
Irr_Offset = 5[ppm]
Clipped = TRUE
Scans = 4096
Total_Scans = 4096
Relaxation_Delay = 2[s]
Recvr_Gain = 58
Temp_Get = 24.2[dC]
X_90_Width = 9.9[us]
X_Acq_Time = 0.69206016[s]
X_Angle = 30[deg]
X_Atn = 8.3[dB]
X_Pulse = 3.3[us]
Irr_Atn_Dec = 25.115[dB]
Irr_Atn_Noe = 25.115[dB]
Irr_Noise = WALTZ
Irr_Pwidth = 76[us]
Decoupling = TRUE
Initial_Wait = 1[s]
Noe = TRUE
Noe_Time = 2[s]
Repetition_Time = 2.69206016[s]

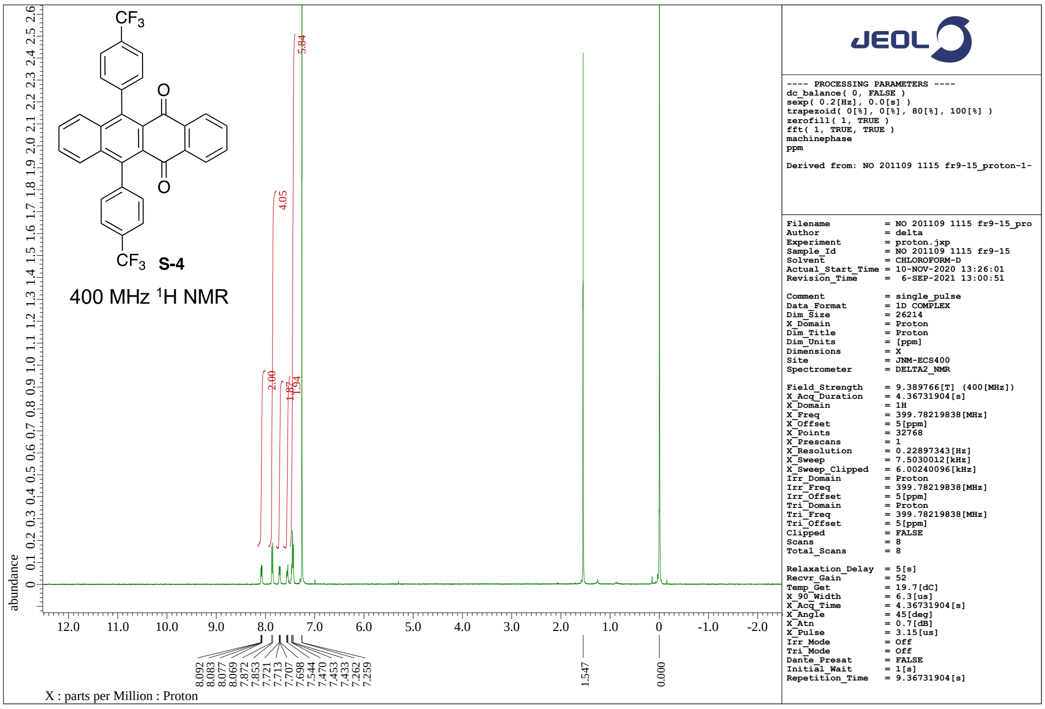
CF3
O
O
CF3 S-4
400 MHz 1H NMR
abundance
X : parts per Million : Proton
JEOL
---- PROCESSING PARAMETERS ----
dc_balance(0, FALSE)
sexp(0.2[Hz], 0.0[s])
trapezoid(0[%], 0[%], 80[%], 100[%])
zerofill(1, TRUE)
fft(1, TRUE, TRUE)
machinephase
ppm
Derived from: NO 201109 1115 fr9-15_proton-1-
Filename = NO 201109 1115 fr9-15_pro
Author = delta
Experiment = proton.jxp
Sample_Id = NO 201109 1115 fr9-15
Solvent = CHLOROFORM-D
Actual_Start_Time = 10-NOV-2020 13:26:01
Revision_Time = 6-SEP-2021 13:00:51
Comment = single_pulse
Data_Format = 1D COMPLEX
Dim_Size = 26214
X_Domain = Proton
Dim_Title = Proton
Dim_Units = [ppm]
Dimensions = X
Site = JNM-ECS400
Spectrometer = DELTA2_NMR
Field_Strength = 9.389766[T] (400[MHz])
X_Acq_Duration = 4.36731904[s]
X_Domain = 1H
X_Freq = 399.78219838[MHz]
X_Offset = 5[ppm]
X_Points = 32768
X_Prescans = 1
X_Resolution = 0.22897343[Hz]
X_Sweep = 7.5030012[kHz]
X_Sweep_Clipped = 6.00240096[kHz]
Irr_Domain = Proton
Irr_Freq = 399.78219838[MHz]
Irr_Offset = 5[ppm]
Tri_Domain = Proton
Tri_Freq = 399.78219838[MHz]
Tri_Offset = 5[ppm]
Clipped = FALSE
Scans = 8
Total_Scans = 8
Relaxation_Delay = 5[s]
Recvr_Gain = 52
Temp_Get = 19.7[dC]
X_90_Width = 6.3[us]
X_Acq_Time = 4.36731904[s]
X_Angle = 45[deg]
X_Atn = 0.7[dB]
X_Pulse = 3.15[us]
Irr_Mode = Off
Tri_Mode = Off
Dante_Presat = FALSE
Initial_Wait = 1[s]
Repetition_Time = 9.36731904[s]

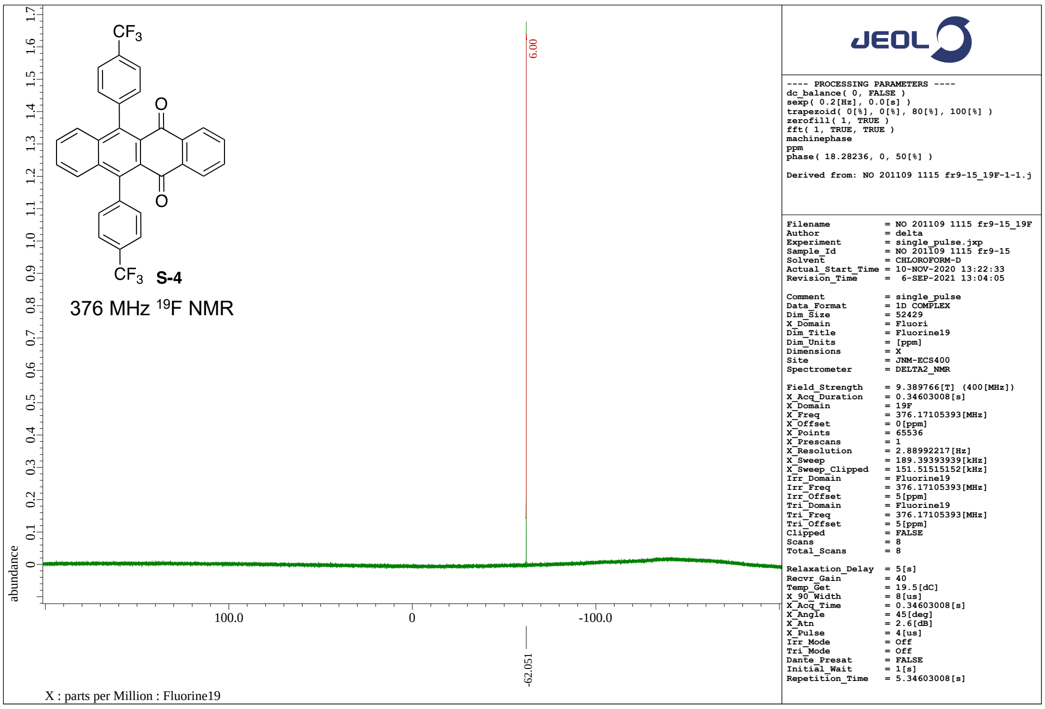
CF3
O
O
CF3 S-4
376 MHz 19F NMR
abundance
X : parts per Million : Fluorine19
JEOL
---- PROCESSING PARAMETERS ----
dc_balance(0, FALSE)
sexp(0.2[Hz], 0.0[s])
trapezoid(0[%], 0[%], 80[%], 100[%])
zerofill(1, TRUE)
fft(1, TRUE, TRUE)
machinephase
ppm
phase(18.28236, 0, 50[%])
Derived from: NO 201109 1115 fr9-15_19F-1-1.j
Filename = NO 201109 1115 fr9-15_19F
Author = delta
Experiment = single_pulse.jxp
Sample_Id = NO 201109 1115 fr9-15
Solvent = CHLOROFORM-D
Actual_Start_Time = 10-NOV-2020 13:22:33
Revision_Time = 6-SEP-2021 13:04:05
Comment = single_pulse
Data_Format = 1D COMPLEX
Dim_Size = 52429
X_Domain = Fluori
Dim_Title = Fluorine19
Dim_Units = [ppm]
Dimensions = X
Site = JNM-ECS400
Spectrometer = DELTA2_NMR
Field_Strength = 9.389766[T] (400[MHz])
X_Acq_Duration = 0.34603008[s]
X_Domain = 19F
X_Freq = 376.17105393[MHz]
X_Offset = 0[ppm]
X_Points = 65536
X_Prescans = 1
X_Resolution = 2.88992217[Hz]
X_Sweep = 189.39393939[kHz]
X_Sweep_Clipped = 151.51515152[kHz]
Irr_Domain = Fluorine19
Irr_Freq = 376.17105393[MHz]
Irr_Offset = 5[ppm]
Tri_Domain = Fluorine19
Tri_Freq = 376.17105393[MHz]
Tri_Offset = 5[ppm]
Clipped = FALSE
Scans = 8
Total_Scans = 8
Relaxation_Delay = 5[s]
Recvr_Gain = 40
Temp_Get = 19.5[dC]
X_90_Width = 8[us]
X_Acq_Time = 0.34603008[s]
X_Angle = 45[deg]
X_Atn = 2.6[dB]
X_Pulse = 4[us]
Irr_Mode = Off
Tri_Mode = Off
Dante_Presat = FALSE
Initial_Wait = 1[s]
Repetition_Time = 5.34603008[s]

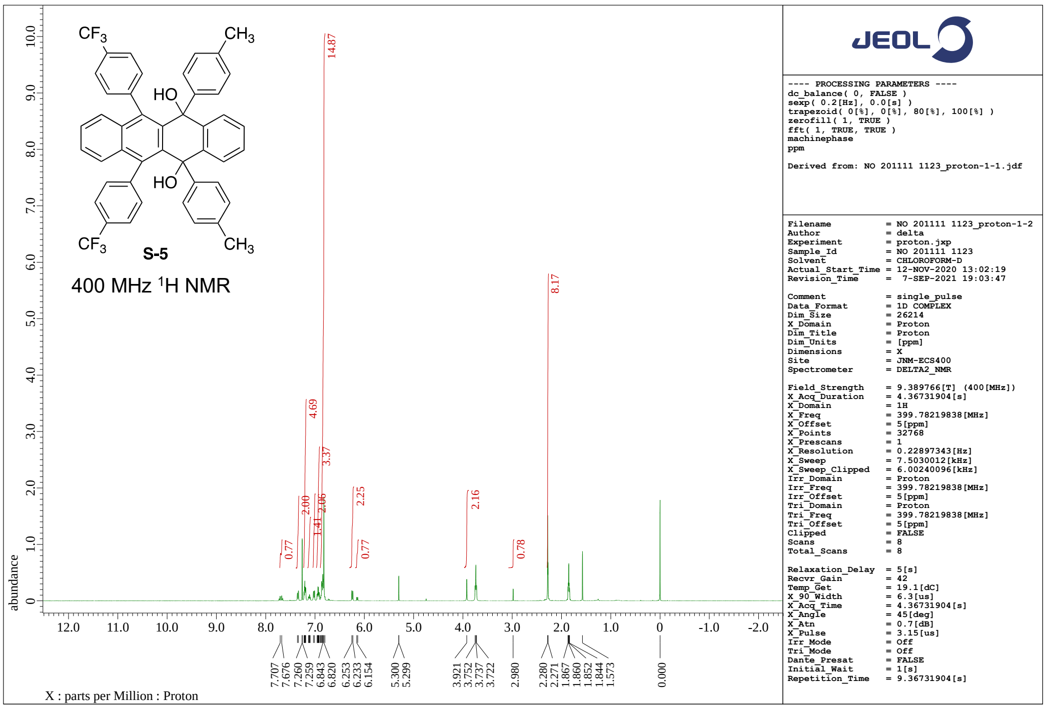
S-5
400 MHz ¹H NMR
abundance
X : parts per Million : Proton
---- PROCESSING PARAMETERS ----
dc_balance(0, FALSE)
sexp(0.2[Hz], 0.0[s])
trapezoid(0[%], 0[%], 80[%], 100[%])
zerofill(1, TRUE)
fft(1, TRUE, TRUE)
machinephase
ppm
Derived from: NO 201111 1123_proton-1-1.jdf
Filename = NO 201111 1123_proton-1-2
Author = delta
Experiment = proton.jxp
Sample_Id = NO 201111 1123
Solvent = CHLOROFORM-D
Actual_Start_Time = 12-NOV-2020 13:02:19
Revision_Time = 7-SEP-2021 19:03:47
Field_Strength = 9.389766[T] (400[MHz])
X_Freq = 399.78219838[MHz]
Scans = 8
Relaxation_Delay = 5[s]

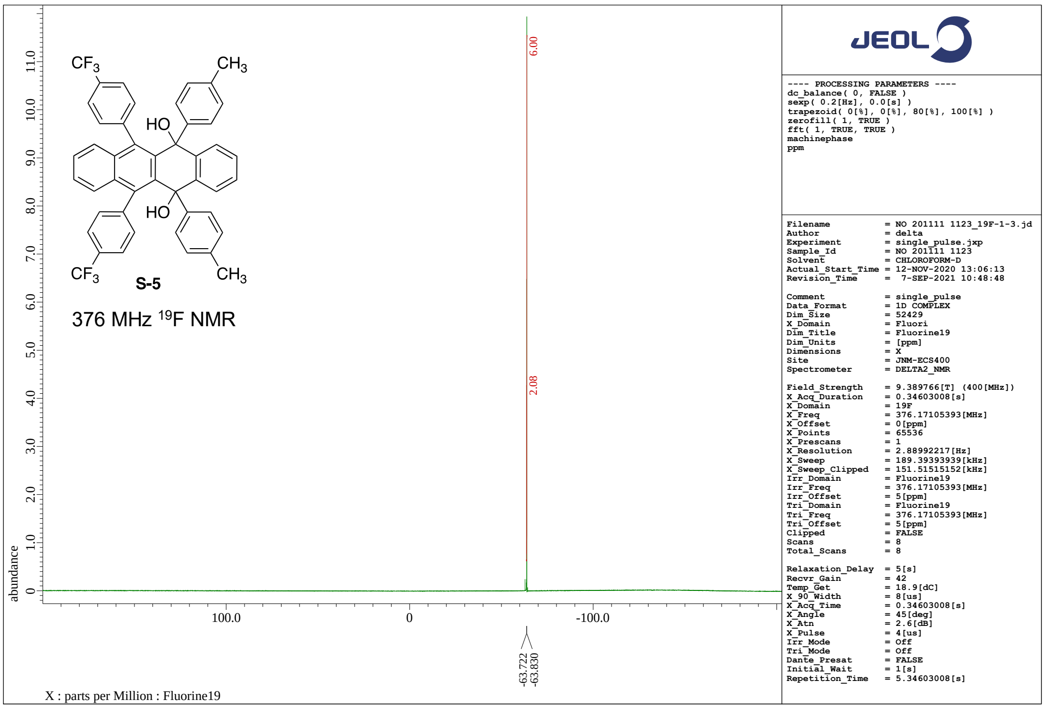
S-5
376 MHz ¹⁹F NMR
abundance
X : parts per Million : Fluorine19
-63.722
-63.830
Filename = NO 201111 1123_19F-1-3.jd
Author = delta
Experiment = single_pulse.jxp
Sample_Id = NO 201111 1123
Solvent = CHLOROFORM-D
Actual_Start_Time = 12-NOV-2020 13:06:13
Revision_Time = 7-SEP-2021 10:48:48
Field_Strength = 9.389766[T] (400[MHz])
X_Freq = 376.17105393[MHz]
Scans = 8
Relaxation_Delay = 5[s]

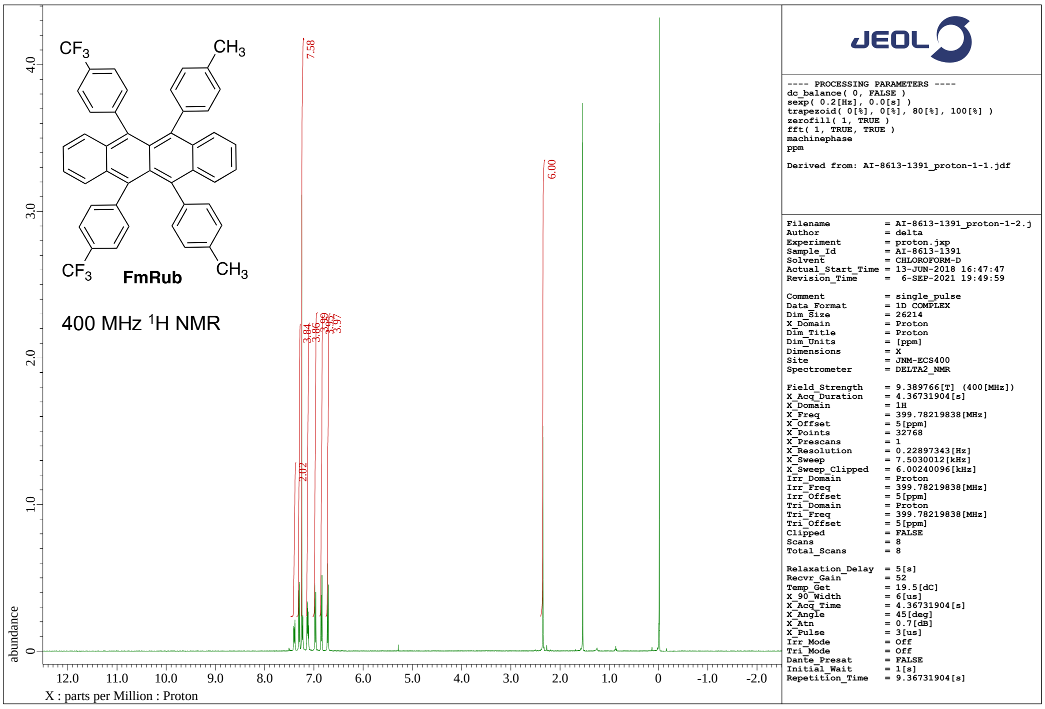
FmRub
400 MHz 1H NMR
CF3
CH3
7.58
6.00
3.84
3.86
3.90
3.98
3.97
2.02
abundance
X : parts per Million : Proton
JEOL
---- PROCESSING PARAMETERS ----
dc_balance(0, FALSE)
sexp(0.2[Hz], 0.0[s])
trapezoid(0[%], 0[%], 80[%], 100[%])
zerofill(1, TRUE)
fft(1, TRUE, TRUE)
machinephase
ppm
Derived from: AI-8613-1391_proton-1-1.jdf
Filename = AI-8613-1391_proton-1-2.j
Author = delta
Experiment = proton.jxp
Sample_Id = AI-8613-1391
Solvent = CHLOROFORM-D
Actual_Start_Time = 13-JUN-2018 16:47:47
Revision_Time = 6-SEP-2021 19:49:59
Comment = single_pulse
Data_Format = 1D COMPLEX
Dim_Size = 26214
X_Domain = Proton
Dim_Title = Proton
Dim_Units = [ppm]
Dimensions = X
Site = JNM-ECS400
Spectrometer = DELTA2_NMR
Field_Strength = 9.389766[T] (400[MHz])
X_Acq_Duration = 4.36731904[s]
X_Domain = 1H
X_Freq = 399.78219838[MHz]
X_Offset = 5[ppm]
X_Points = 32768
X_Prescans = 1
X_Resolution = 0.22897343[Hz]
X_Sweep = 7.5030012[kHz]
X_Sweep_Clipped = 6.00240096[kHz]
Irr_Domain = Proton
Irr_Freq = 399.78219838[MHz]
Irr_Offset = 5[ppm]
Tri_Domain = Proton
Tri_Freq = 399.78219838[MHz]
Tri_Offset = 5[ppm]
Clipped = FALSE
Scans = 8
Total_Scans = 8
Relaxation_Delay = 5[s]
Recvr_Gain = 52
Temp_Get = 19.5[dC]
X_90_Width = 6[us]
X_Acq_Time = 4.36731904[s]
X_Angle = 45[deg]
X_Atn = 0.7[dB]
X_Pulse = 3[us]
Irr_Mode = Off
Tri_Mode = Off
Dante_Presat = FALSE
Initial_Wait = 1[s]
Repetition_Time = 9.36731904[s]

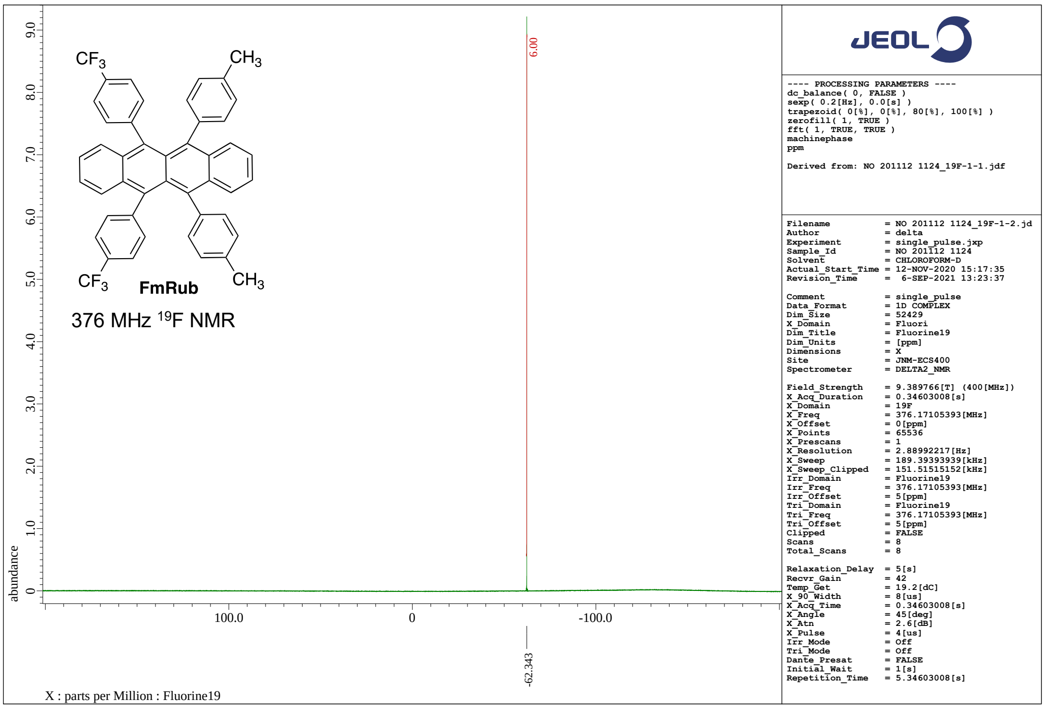
FmRub
376 MHz 19F NMR
CF3
CH3
6.00
-62.343
abundance
X : parts per Million : Fluorine19
JEOL
---- PROCESSING PARAMETERS ----
dc_balance(0, FALSE)
sexp(0.2[Hz], 0.0[s])
trapezoid(0[%], 0[%], 80[%], 100[%])
zerofill(1, TRUE)
fft(1, TRUE, TRUE)
machinephase
ppm
Derived from: NO 201112 1124_19F-1-1.jdf
Filename = NO 201112 1124_19F-1-2.jd
Author = delta
Experiment = single_pulse.jxp
Sample_Id = NO 201112 1124
Solvent = CHLOROFORM-D
Actual_Start_Time = 12-NOV-2020 15:17:35
Revision_Time = 6-SEP-2021 13:23:37
Comment = single_pulse
Data_Format = 1D COMPLEX
Dim_Size = 52429
X_Domain = Fluori
Dim_Title = Fluorine19
Dim_Units = [ppm]
Dimensions = X
Site = JNM-ECS400
Spectrometer = DELTA2_NMR
Field_Strength = 9.389766[T] (400[MHz])
X_Acq_Duration = 0.34603008[s]
X_Domain = 19F
X_Freq = 376.17105393[MHz]
X_Offset = 0[ppm]
X_Points = 65536
X_Prescans = 1
X_Resolution = 2.88992217[Hz]
X_Sweep = 189.39393939[kHz]
X_Sweep_Clipped = 151.51515152[kHz]
Irr_Domain = Fluorine19
Irr_Freq = 376.17105393[MHz]
Irr_Offset = 5[ppm]
Tri_Domain = Fluorine19
Tri_Freq = 376.17105393[MHz]
Tri_Offset = 5[ppm]
Clipped = FALSE
Scans = 8
Total_Scans = 8
Relaxation_Delay = 5[s]
Recvr_Gain = 42
Temp_Get = 19.2[dC]
X_90_Width = 8[us]
X_Acq_Time = 0.34603008[s]
X_Angle = 45[deg]
X_Atn = 2.6[dB]
X_Pulse = 4[us]
Irr_Mode = Off
Tri_Mode = Off
Dante_Presat = FALSE
Initial_Wait = 1[s]
Repetition_Time = 5.34603008[s]

S2. Atomic force microscopy (AFM) images of the fmRub/RubSC sample

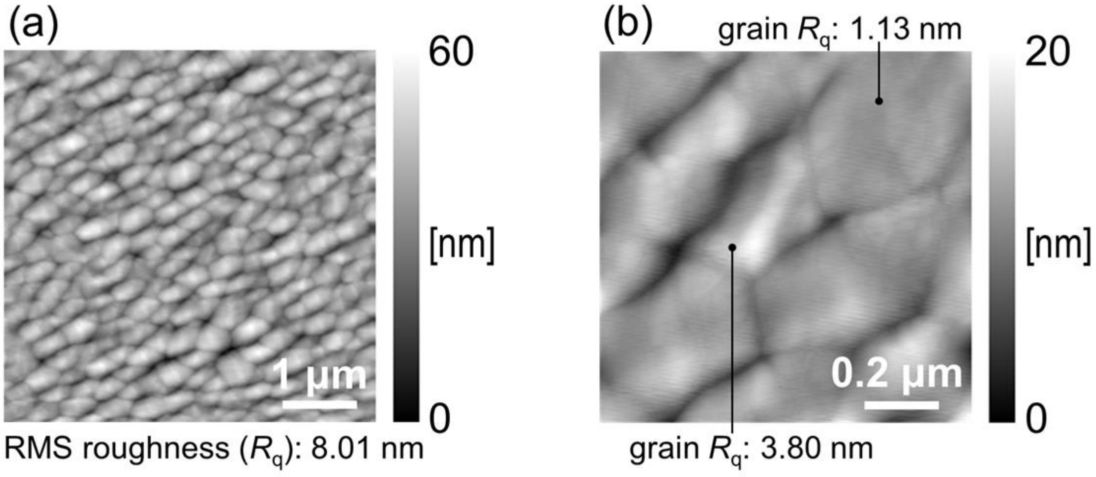

Fig. S3: (a) 5 µm × 5 µm AFM image of a RubSC sample covered with a 50 nm-thick fmRub overlayer. The root-mean-square roughness (R_q) of the whole image area is 8.01 nm. (b) A close-up (1 µm × 1 µm) AFM image of the image (a). The R_q of two representative grains in the image are also given.

S3. Crystal structures of rubrene and bis(trifluoromethyl)-dimethyl-rubrene

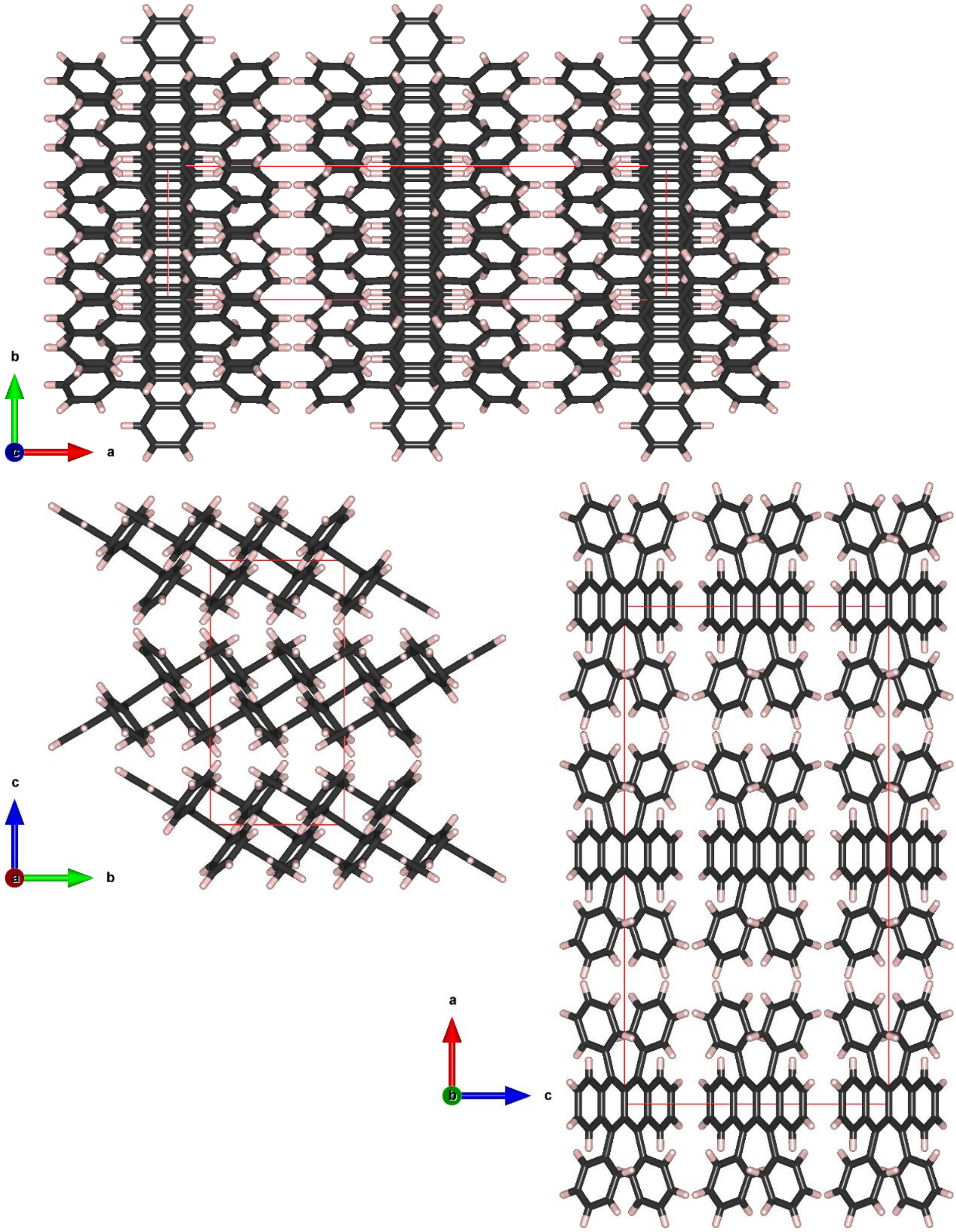

Fig. S2-1: Crystal structure and molecular arrangement of rubrene drawn by VESTA[3] based on a reported crystallographic information file[4]. The unit cell is displayed in red rectangles.

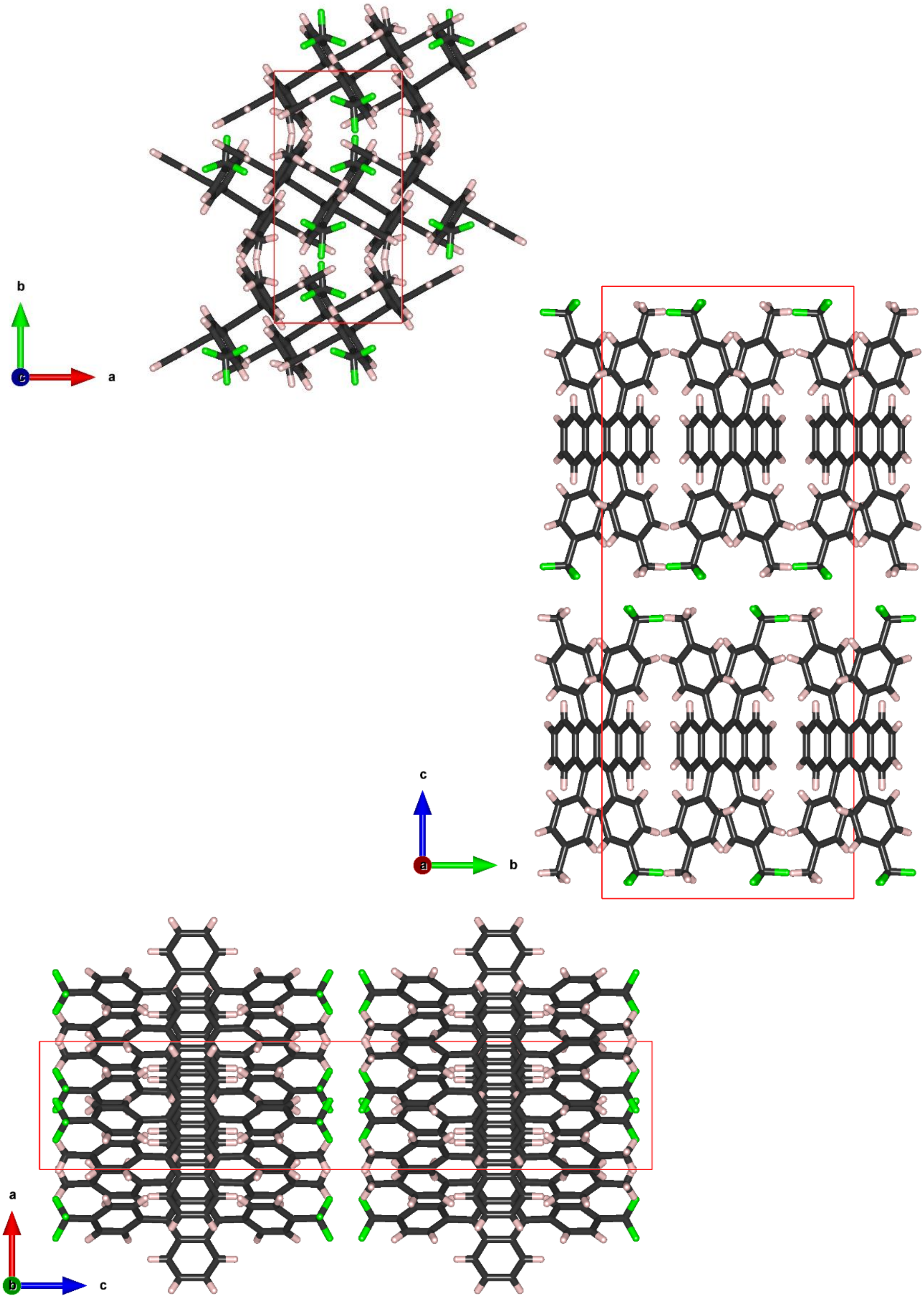

Fig. S2-2: Crystal structure and molecular arrangement of bis(trifluoromethyl)-dimethyl-rubrene drawn by VESTA[3] based on a reported crystallographic information file[2]. The unit cell is displayed in red rectangles.

S4. Peak separation for the q_{xy} profiles of fmRub 102 and 022 spots

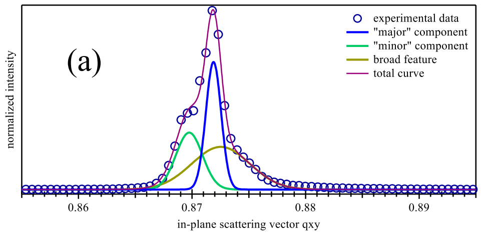

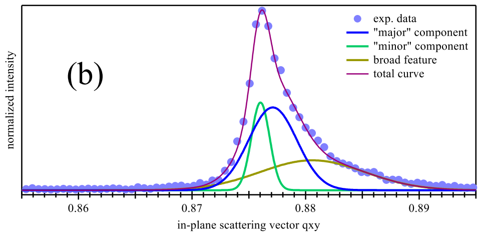

Fig. S4: The least-squares fitting results for (a) fmRu 022 and (b) fmRub 102 diffraction profiles.

Reference

1) Yagodkin, E.; Xia, Y.; Kalihari, V.; Frisbie, C. D.; Douglas C. J. Synthesis, Solid State Properties, and Semiconductor Measurements of 5,6,11,12-Tetrachlorotetracene. *J. Phys. Chem. C* **2009**, *113*, 16544–16548.

2) McGarry, K. A.; Xie, W.; Sutton, C.; Risko, C.; Wu, Y.; Young Jr., V. G.; Brédas, J.-L.; Frisbie, C. D.; Douglas, C. J. Rubrene-Based Single-Crystal Organic Semiconductors: Synthesis, Electronic Structure, and Charge-Transport Properties. *Chem. Mater.* **2013**, *25*, 2254–2263.

3) Momma, K. A.; Izumi, F. VESTA 3 for Three-Dimensional Visualization of Crystal, Volumetric and Morphology Data. *J. Appl. Cryst.* **2011**, *44*, 1272–1276.

4) Jurchescu, O. D.; Meetsma, A.; Palstra, T. T. M. Low-Temperature Structure of Rubrene Single Crystals Grown by Vapor Transport. *Acta Cryst. B* **2006**, *62*, 330–334.
